# Combined somatic mutation and transcriptome analysis reveals region-specific differences in clonal architecture in human cortex

**DOI:** 10.1016/j.celrep.2025.116458

**Published:** 2025-11-14

**Authors:** Vinayak V. Viswanadham, Sonia N. Kim, Emre Caglayan, Ryan N. Doan, Yanmei Dou, Sara Bizzotto, Sattar Khoshkhoo, August Yue Huang, Rebecca Yeh, Brian H. Chhouk, Alex Truong, Kathleen M. Chappell, Marc Beaudin, Alison Barton, Shyam K. Akula, Yifan Zhao, Lariza Rento, Michael Lodato, Ryan A. Szeto, Javier Ganz, Pengpeng Li, Jessica W. Tsai, Robert Sean Hill, Peter J. Park, Christopher A. Walsh

**Affiliations:** 1 Department of Biomedical Informatics, Harvard Medical School, Boston, MA, USA; 2 Bioinformatics and Integrative Genomics Program, Harvard Medical School, Boston, MA, USA; 3 Division of Genetics and Genomics, Manton Center for Orphan Disease Research, Department of Pediatrics, and Howard Hughes Medical Institute, Boston Children’s Hospital, Boston, MA 02115, USA; 4 Departments of Pediatrics and Neurology, Harvard Medical School, Boston, MA 02115, USA; 5 Broad Institute of MIT and Harvard, Cambridge, MA 02142, USA; 6 Program in Biological and Biomedical Sciences, Harvard University, Boston, MA, USA; 7 Department of Neurology, Brigham and Women’s Hospital, Boston, MA, USA; 8 Research Computing, Harvard Medical School, Boston, MA, USA; 9 Program in Health Sciences & Technology, Harvard Medical School & Massachusetts Institute of Technology, Boston, MA, USA; 10 Present address: Department of Neurosurgery, Stanford University School of Medicine, Stanford, CA; 11 Present address: Department of Molecular, Cell and Cancer Biology, Genome Integrity Program, University of Massachusetts Chan Medical School, Worcester, MA, USA; 12 Present address: Pediatric Neurology Program, Boston Medical Center, Boston, MA; 13 Present address: Children’s Hospital Los Angeles, Cancer and Blood Disease Institute and Saban Research Institute; Keck School of Medicine of University of Southern California, Department of Pediatrics; USC Norris Comprehensive Cancer Center, Epigenetic Regulation in Cancer Program, Los Angeles, CA; 14 These authors contributed equally; 15 Lead contact

## Abstract

The human cerebral cortex is specialized into regions, but little is known about how human cellular lineages shape cortical regional variation and neuronal cell-type distribution during development. Here, we map single-cell lineages of human cortical regions and neuronal subtypes using >1,000 somatic single-nucleotide variants (sSNVs) identified from deep bulk whole-genome sequencing and analyzed over 25 regions and >72,000 single cells. In the fronto-parietal cortex, sSNVs are rarely restricted, marking neuron-generating clones that disperse into neighboring regions. In contrast, the primary visual cortex harbors 30%–70% more sSNVs than the neighboring secondary visual cortex. Clones at this border exhibit more restricted dispersion, suggesting late developmental lineage segregation. Single-nucleus sSNV and whole-transcriptome analysis reveal glutamatergic neuron clones with modest regional restrictions that share low-mosaic sSNVs with some GABAergic neurons, suggesting a recent dorsal cortical progenitor. Our analysis reveals human-specific cortical lineage patterns, regional differences in clonal patterns, and late divergence of some glutamatergic/GABAergic lineages.

## In brief

Viswanadham, Kim, et al. combine somatic mutational and transcriptome analyses to trace the lineages of neuronal clones in the human cerebral cortex. They explore the differences between the visual and prefrontal cortex in clonal development, dispersion, and identities and dissect the lineages of late-rising cortical glutamatergic and GABAergic neurons.

## Graphical Abstract

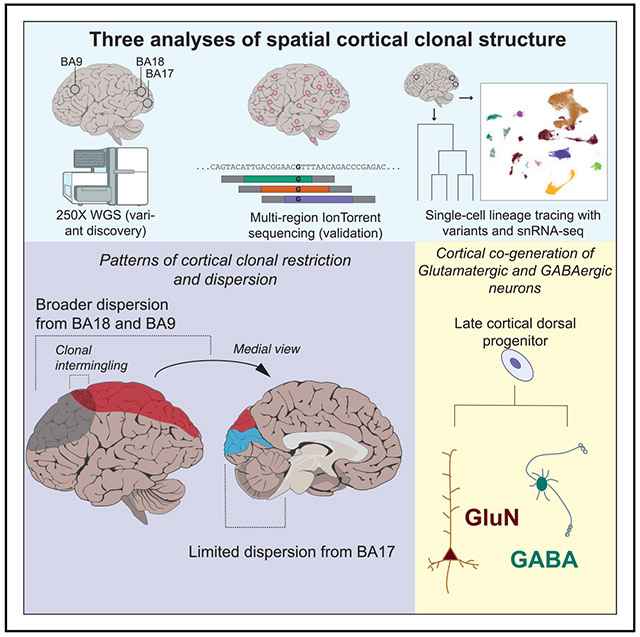

## INTRODUCTION

Pattern formation in the human cerebral cortex has long fascinated neuroscientists. For over a century, Brodmann area (BA) designations have encapsulated differences in the spatial patterns of cell bodies and myelin fibers across human cortical areas. Magnetic resonance imaging (MRI) of function and connectivity in large human cohorts has largely confirmed correlations between BAs and functional specializations and validated their relevance to complex cognitive and behavioral tasks.^1,2^

Diverse cortical areas arise by a complex interplay of patterning forces. Early regional specification of the cortex does not explicitly require axonal input^3^; instead, it involves gradients of secreted factors and transcription factors.^4^ On the other hand, thalamic afferents regulate aspects of cell identity^4-7^ and progenitor proliferation.^8^ In macaque monkeys, prenatal removal of visual thalamic inputs causes a reduced extent of the primary visual cortex (BA17), along with a shift of the unique histologically identifiable boundary that separates the primary and secondary visual cortex (BA18).^9-11^

Studies in mice suggest an important role for cell lineage in regional and cell-type specification. Radial glia, which produce glutamatergic neurons (GluNs; corresponding to excitatory) of the cortex, produce large clones of neurons distributed from superficial to deep layers that are preferentially connected to each other,^12-14^ potentially sharing physiological properties.^15-17^ In contrast, other studies suggest stochasticity in GluN clones, with pyramidal neuron clones showing a wide range of sizes and laminar configurations, including deep-layer-restricted cortical lineages.^14,18^ GABAergic neurons (GABNs; corresponding to inhibitory) for the cerebral cortex are mainly generated outside the cortex, in the ganglionic eminences (GEs),^19-21^ and show widespread clonal dispersion across the cortex.^22-27^

The discovery that somatic mutations occur widely during human development has enabled direct cell lineage and clonal analysis of postmortem samples from single-cell or bulk DNA sequencing.^28-30^ Recent studies have largely focused on the fronto-parietal cortex and suggest widespread cortical dispersion of neuronal clones consisting of >1%–3% of cells,^30-34^ though the possibility of more limited dispersion of later, smaller clones remains.^29^ These studies are challenged by technical difficulties in simultaneously analyzing somatic single-nucleotide variants (sSNVs) and transcriptional markers of cell type.

Here, we provide the first simultaneous analysis of patterns of clonal dispersion, coupled to both targeted and full 10× Genomics analysis of cell type. We confirm earlier reports of widespread clonal dispersion across the frontal cortex, with even late-rising mosaic variants present in GluNs across multiple regions. However, we describe here regional inhomogeneity in clonal structure superimposed on this dispersion at the BA17/18 border. We also observed the frequent co-generation of GluNs and GABNs at late stages of neurogenesis^33,35,36^ and provide the first description of the detailed neuronal subtypes found in these shared clones *in vivo*.

## RESULTS

### Primary visual cortex harbors more sSNVs at low-mosaic fractions than secondary visual cortex

**Figure 1.**
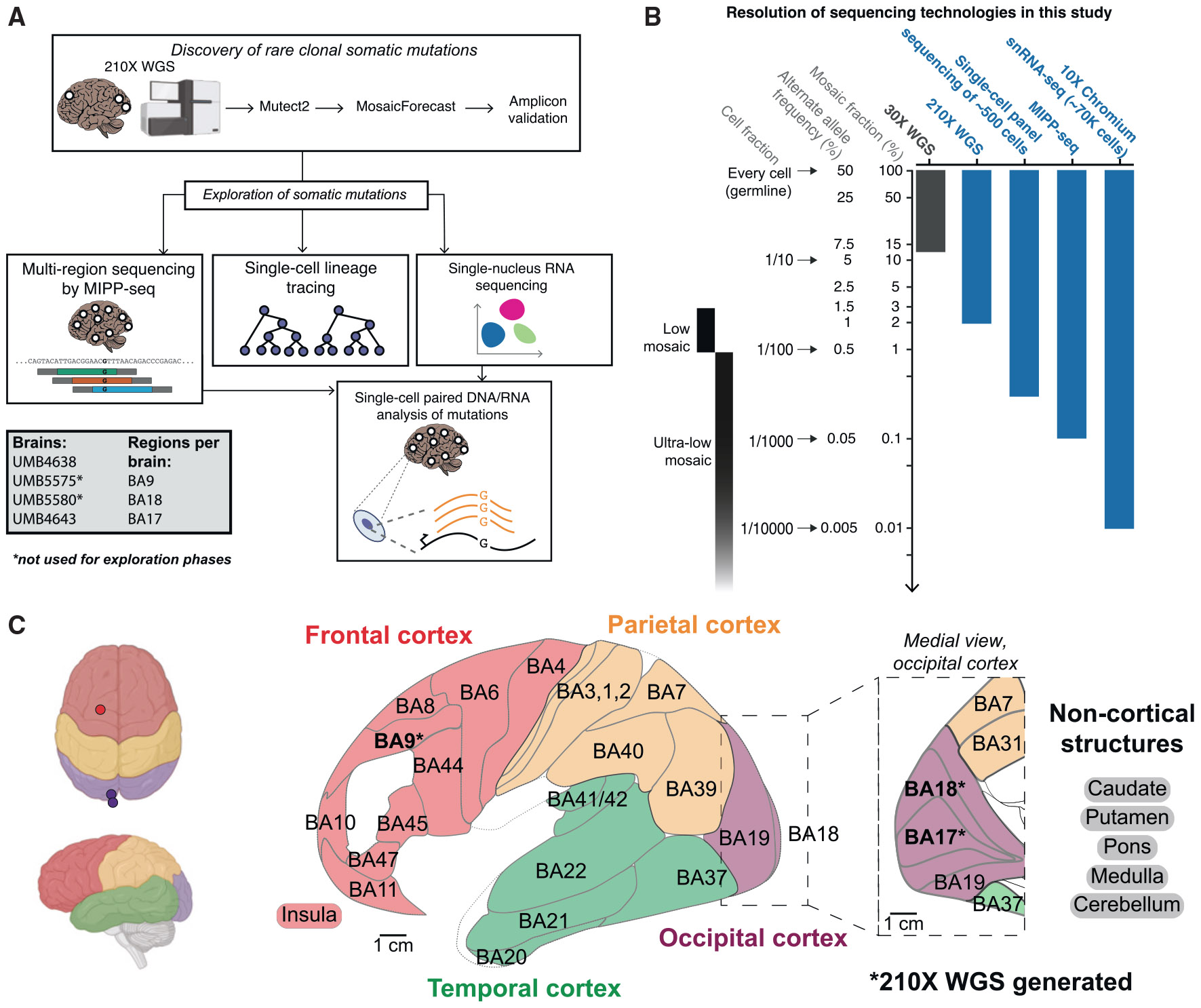
Study design (A) Outline of the study. (B) A schematic comparison of the resolution into mosaic (cell) fractions of different technologies in this study (shaded in blue). (C) The Brodmann areas and brain structures that were sampled for studies of the spread of sSNVs. BA17, BA18, and BA9 were sampled for WGS, snRNA-seq, and MIPP-seq, while the remaining cortical regions shown were sampled for MIPP-seq only. Sampled regions are colored by the lobe of origin. Regions separated by slashes (BA3/1/2 and BA41/42) were studied together. The medial view shows the surface of the hemisphere facing inward toward the other hemisphere.

First, to generate informative clonal markers, we identified and validated clonal mosaic sSNVs from BA9 within the prefrontal cortex (PFC), BA17 (primary visual cortex), and BA18 (secondary visual cortex) (Figures 1A and S1A; Table S1.1; STAR Methods). We performed whole-genome sequencing (WGS) to 210× coverage on bulk DNA from four neurotypical individuals. Using MosaicForecast,^37,38^ we detected 984 sSNVs with a false discovery rate of 5%, adding to sSNVs discovered in some samples in prior studies.^31^ Our WGS analysis identifies sSNVs present at a ≥2% mosaic (cell) fraction (MF), equivalent to alternate allele fractions (AAFs) of ≥1%, with sensitivity that varies with the MF (Table S1.7; MF is estimated to be twice the AAF) (Figure 1B).

**Figure 2.**
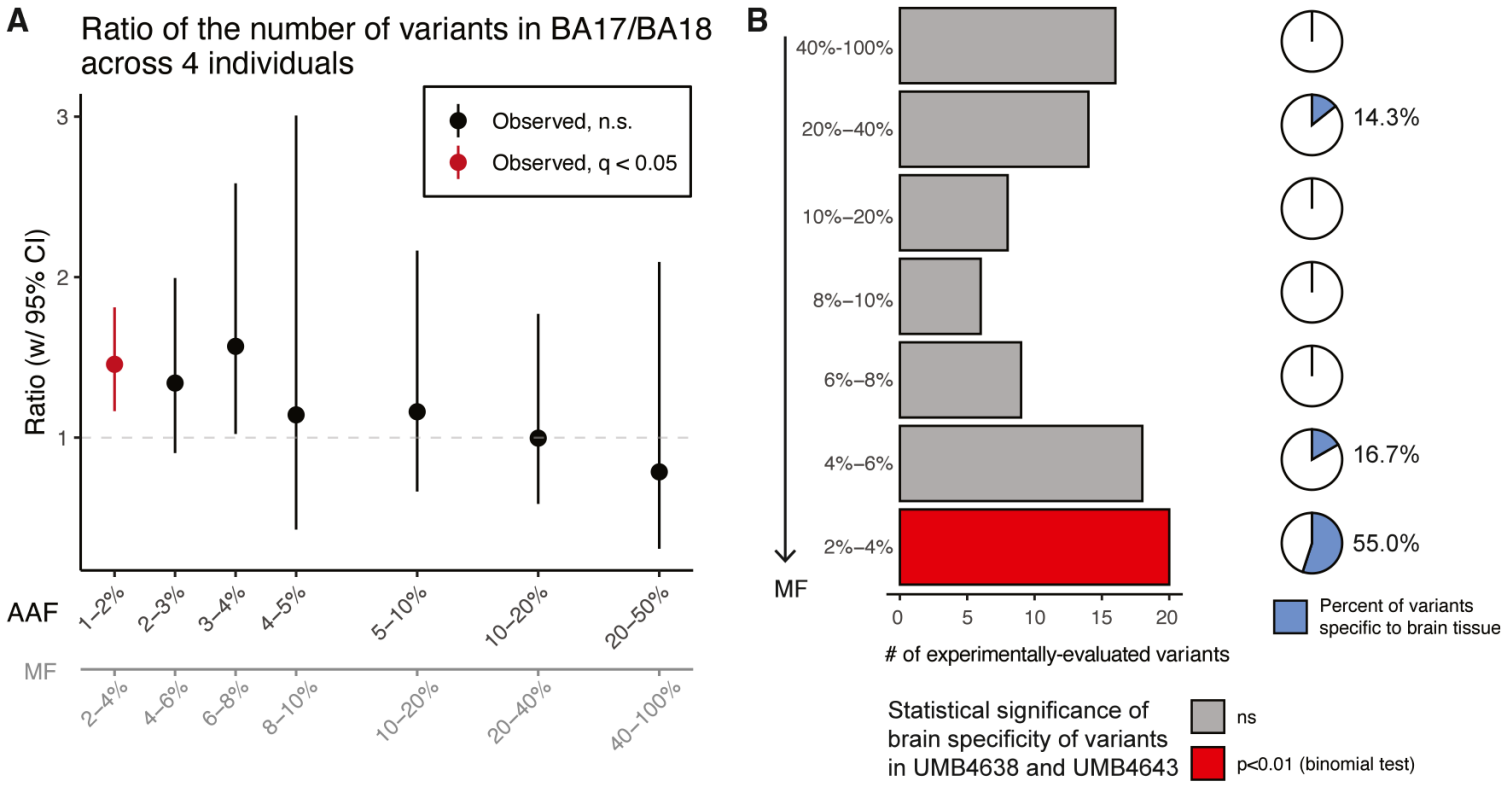
Regional differences in the number and mosaicism of sSNVs within the visual cortex (A) Ratio of the number of somatic mutations in each alternate allele frequency (AAF) and corresponding mosaic fraction (MF) range between the adjacent regions in the visual cortex (BA18 and BA17) across all 4 donors subjected to 210× WGS (STAR Methods). Significance evaluated at q < 0.05. 95% CI for ratios are shown. (B) Subset of amplicon-validated sSNVs present in tissues derived from major germ layers. Significance evaluated at q < 0.01. Embryonic germ layer data were obtained from Bizzotto et al.^31^

Across all four individuals, BA17 consistently showed more sSNVs than adjacent BA18, with BA9 also exceeding BA18 in 3 out of 4 samples (Figures S3A-S3C; Table S1.2). We estimated the ratio of sSNVs detectable in BA17 versus BA18 at different AAFs after controlling for sensitivity (STAR Methods; Table S1.7). Strikingly, BA17 contains 30%–70% more sSNVs than BA18 at a 2%–4% MF (Figure 2A) (empirical *p* < 0.05; 95% confidence interval [CI]: 1.16–1.81 for observed versus 0.85–1.16 for expected). In contrast, sSNVs at a >20% MF were distributed equally in all three regions (Figures 2A and S3C), likely arising in early embryonic cell divisions before the formation of the brain.^31^ Although the mean MFs of BA17-restricted sSNVs were not different from those of BA18-specific sSNVs (~4%), the MFs of regionally restricted sSNVs were lower than those of shared sSNVs (15%–25%) (*p* = 7.32e–3 in BA17 and *p* = 5.47e–3 in BA18, two-sided *t* test; Figure S3D).

Amplicon sequencing (>10,000×; STAR Methods) on 155 sSNVs from BA17 and/or BA18 confirmed the >1.5× higher number of sSNVs in BA17 compared to BA18 (Tables S1.3 and S1.6). We validated 138 (89%) of 155 sSNVs. Most (>2/3) sSNVs at a <4% MF were specific to the brain (Figure 2B; Table S1.4) (*p* < 0.05, chi-squared test), consistent with many brain-restricted SNVs appearing at a 2%–10% MF.^31^ Our overall sSNV validation rate is in line with MosaicForecast’s expected performance.^37,38^

**Figure 3.**
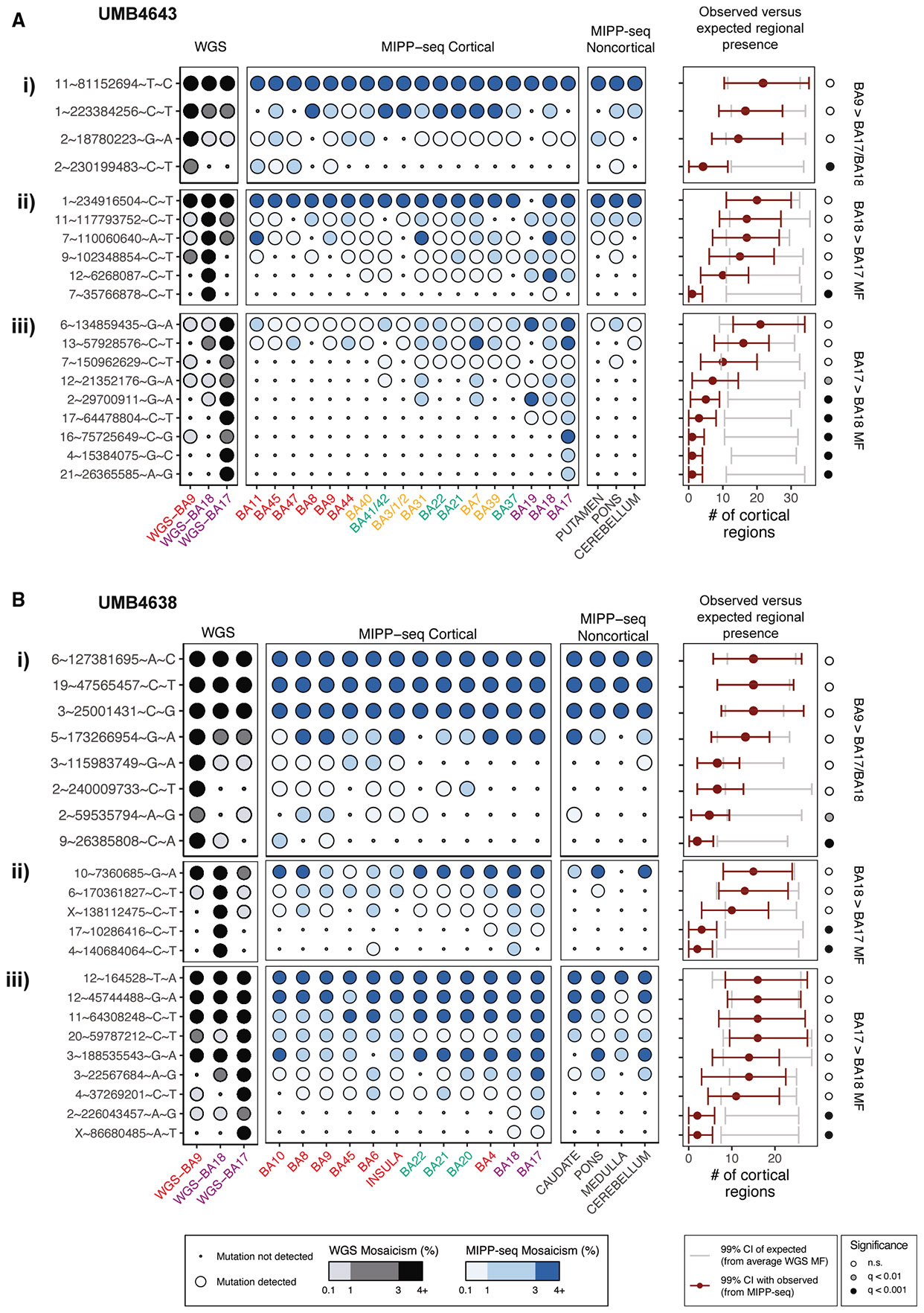
Spatial distributions of sSNVs across cortical and non-cortical structures The presence of sSNVs selected for MIPP-seq in UMB4643 (A) and UMB4638 (B) with greater MFs in BA9 versus BA17 and BA18 (i), BA18 versus BA17 (ii), or vice versa (iii) are shown across multiple cortical regions. Far left: MFs of sSNVs in WGS regions. Middle left: spatial map of sSNVs in regions analyzed by MIPP-seq is shown. In individual WGS and MIPP-seq heatmaps, tissues are arranged (left to right) in anterior to posterior cortical section ordering, and their labels are colored based on the scheme in Figure 1C. Mutations are vertically arranged from broadest to least present across the tissues. The mosaic fractions of an sSNV in a region are colored by range: 0.1%–1% (“ultra-low”), 1%–3% (“low”), and 3%–4+%. Middle right: 99% Poisson CIs indicate the number of regions in which a mutation is expected (gray) or observed (dark red) to occur (STAR Methods). Far right: *q* values indicate the statistical significance of the number of regions that are observed to harbor the sSNV.

Multiple independent primer PCR sequencing (MIPP-seq) analysis of many cortical regions confirmed that sSNVs with MF differences across the BA17/BA18 border showed distinct cortex-wide patterns (Figure 3). To assess the significance of an sSNV’s regional distribution, we used its averaged cortex-wide MFs from WGS (i.e., if the sSNV were “uniformly mixed” in the cortex) to simulate the expected number of sSNV-harboring regions (STAR Methods). In UMB4643 (Figure 3Aiii; Table S2.2), 6/9 BA17>BA18 (the MF in BA17 is higher than in BA18) sSNVs tested were present in a statistically significantly smaller number of regions than expected from its WGS MF (*q* < 0.01 for 1 sSNV and *q* < 0.001 for 5 sSNVs, Poisson *p* values), with 3/6 significantly restricted BA17>BA18 sSNVs detected only in BA17 (1.1%–3.1%; chr16:75725649, chr4:15384075, and chr21:26365585). Without the influence of cortical structure, variants at similar MFs would be detected in 20 regions. In contrast, only 1/6 BA18>BA17 sSNVs (Figure 3Aii) (chr7:35766878) showed significant restriction to 1 region (BA18; *q* < 0.001) and at an ultra-low MF (0.77%). Regional restriction in MIPP-seq occurs for clones at MFs <4% overall, consistent with the trend in WGS that regional differences between BA17 and BA18 are apparent for sSNVs at or <4% MFs.

Even those sSNVs not strictly limited to one region showed different dispersion patterns based on their MFs across the BA17/BA18 border. In UMB4638 (Figures 3Bii and 3Biii; Table S2.1), both BA17>BA18 sSNVs were significantly restricted to the visual cortex (BA17 and BA18), while both BA18>BA17 sSNVs were detected at ultra-low MFs in the frontal lobe (BA4 and BA6). In both individuals (Figures 3Aii, 3Aiii, 3Bii, and 3Biii), BA17>BA18 sSNVs found in significantly fewer regions than expected were restricted to more posterior regions of the cortex, often not crossing past BA18 or the posterior edge of the adjoining parietal lobe (BA31 in UMB4643). However, BA18>BA17 sSNVs at similar MFs were dispersed as far as the anterior regions of the cortex, such as the anterior surfaces of the temporal lobe (BA21 and BA22 in UMB4643) and the frontal cortex (BA4 and BA6 in UMB4638) at low or ultra-low MFs.

In both individuals, clonal restriction or asymmetric dispersion across the BA17/BA18 border contrasts with sSNVs in the frontal cortex, which disperse across multiple regions even at MFs <1% (Figures 3Ai and 3Bi). We observed that none of the low- or ultra-low BA9-localized mutations in either individual that appear at greater MFs than in BA17 or BA18 (BA9>BA17/BA18) appear restricted to a single region, instead appearing at comparable MFs in adjacent regions and crossing over into other lobes. We and others have previously described this dispersion pattern in the frontal cortex.^30-33^ However, the BA17/BA18 border may represent a significant exception to adjacent cortical territories whose clones remain robustly localized with asymmetric cross-boundary presence.

Taken together, our data suggest a fundamental difference in the clonal structures of the visual cortex at MFs ≤4% (Figure 2A) that contrasts with clonal dispersion patterns seen in the frontal lobe. BA17 harbors more sSNVs with a ≤4% MF than BA18, indicating greater regional restriction, with regionally restricted sSNVs in BA17 appearing at higher MFs than similar sSNVs in BA18 (1%–3% versus <1%, Figures 3Aii and 3Aiii). These patterns are consistent with reports of the higher neuronal density^39,40^ and proliferation^8,41-43^ in the incipient and adult primary versus secondary visual cortex in non-human primates, as well as constrained radial migration across the border during development. Studies of non-human primates also suggest that the proliferation rate is greater for progenitors underlying the incipient primary versus secondary visual cortex.^44,45^ We also hypothesize that the 4% threshold may mark the start of brain-specific developmental events, given that BA17- or BA18-restricted clones’ MFs do not exceed 4% and become primarily brain restricted.

### Inferring the timing of cortical patterning using single-cell lineage tracing

We defined single-cell lineage trees by genotyping 122 brain-restricted sSNVs in 1,131 single NeuN+ (neurons) and NeuN− (non-neuronal) cells from BA17, BA18, and BA9 across UMB4638 and UMB4643 (STAR Methods; Figures S4A-S4E; Table S3). Inspired by progress in hematopoietic stem cell lineages,^46^ we fitted a coalescent model to our neuronal data to compute “coalescent times” of cells sharing clonal sSNVs. Coalescent times estimate when a subpopulation of cells sharing a particular variant diverged from its most recent common ancestor (MRCA), thus also estimating the variant’s time of origin (TOO) within the MRCA. Using published estimates for the number of new cells generated during human fetal neurogenesis,^47^ we can convert each variant’s coalescent time (in units of generations) to an estimate of each variant’s TOO as the post-MRCA week (PMW), which is the number of weeks that have elapsed since the MRCA of the entire corresponding lineage tree.

**Figure 4.**
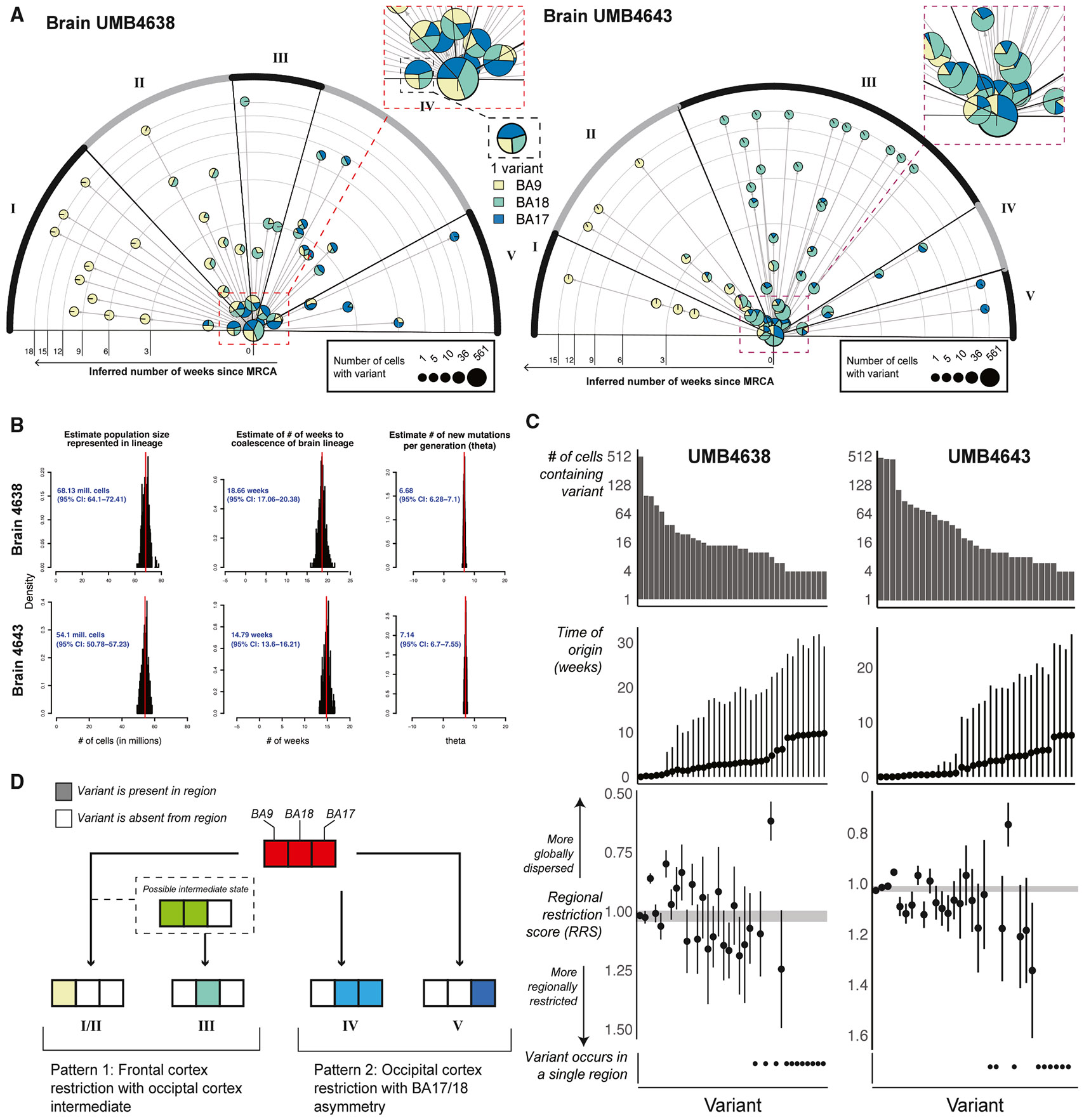
Lineage and coalescent inference of the timing and patterns of clonal allocation across the visual and prefrontal cortex (A) Radial plot showing the time of origin (TOO) of variants inferred by a coalescent model applied to single-cell genomic lineages. Each pie chart corresponds to a variant, sized by the number of cells carrying the variant and sliced by the proportion of cells found in each region. Arrows are drawn between variants that occur on consecutive lineage tree branches. Rings on the radial plot correspond to time in weeks. Variants are arranged in different sectors (I–V), each of which is determined by the overall regional identity of the cells carrying the variants (variants with more anterior distributions are placed on the left). The angles of the arrows in each sector are placed arbitrarily and spaced out for visual clarity. For visual clarity, the insets of the radial plots show some of the early-rising variants. (B) Posterior distributions of coalescent model parameters. (C) Associations between estimated TOO and regional restriction, as quantified by the regional restriction statistic (RRS) for each variant found in 2 or more cells in its corresponding lineage tree. From top to bottom: the number of cells carrying each variant, the TOO estimates (in weeks) with 95% credible intervals, and the RRS computed for each variant (see STAR Methods). The RRS range for germline variants is plotted as the gray band encompassing RRS = 1 as a reference. Confidence intervals were constructed from bootstrapped estimates of RRS taken from sampling cells in clades of the lineage tree. (D) A schematic of the two main patterns that hypothesize how variants (each belonging to a different sector) are dispersed across the cortex based on the lineage analysis.

Analyzed neurons formed over ~16 weeks (Figures 4A and 4B), corresponding roughly to the span of human cortical neurogenesis from gestational weeks (GW) 10–25^48,49^. If MRCAs first arose near the start of neurogenesis (i.e., PMW0 = GW10), then our lineages would include developmental events throughout most of the neurogenesis. We further estimated that our cells were sampled from a population of ~60 million cells harboring mutations studied in each tree, with ~7 new mutations arising in the population during each generation (Figure 4B). sSNVs marked clones of diverse sizes (Figure S4E), and 2–9 sSNVs distinguished each cell (Figure S4F).

Combining the lineage and spatial information enables investigation into regional genealogies of cells carrying measured sSNVs. We constructed timelines of sSNVs that tracked the spread of cortical clones (Figure 4A; STAR Methods). By conducting *k*-means clustering on the matrix of sSNV by MF (estimated by fractions of single cells) in each region, we identified five modes of spatial dispersion (STAR Methods; Table S3.21). First, 18 variants across the two brains are restricted to BA9 by PMW2 (cluster I), representing a subset of clones isolated to the PFC early in neurogenesis. Second, 22 variants end up primarily within occipital cortex, either mostly restricted to BA17 (cluster V) or split across BA17 and BA18 (cluster IV) by PMW4–6. Nine of these 22 occipital variants were also detected at low MFs in BA9 at or before PMW6, suggesting that the exclusion of these variants from the frontal cortex is not complete before then. Clusters I, IV, and V represent the allocation of clones between the occipital and prefrontal cortices at different times. Finally, the remaining variants represent the exclusion of clones away from BA17. We observed 41 variants excluded from BA17 over PMW1–3 that ended up restricted to either BA18 by PMW6 (cluster III) or to BA9 by PMW9 (cluster II) separately from and more gradually than the early-BA9 variants (cluster I). Given that cells in both clusters are largely restricted to BA9, cluster II could represent a BA18/9 intermediate of cluster I but would require further experimental confirmation.

We quantified the change in regional restriction with a “regional restriction statistic” (RRS) (STAR Methods; Table S3.21). Over time and at increasingly lower MFs, newly arising sSNVs are restricted to one of the three regions (RRS > 1) (Figure 4C). The exceptions were two low-mosaic sSNVs arising between PMW5 and PMW10 that were distributed across two of the regions and depleted in one region (RRS < 1; one across BA18/9 and the other across BA17/18).

MIPP-seq added granularity to our single-cell lineages. First, MIPP-seq supported the prediction that BA18>BA17 clones would also have more anterior final destinations than BA17>BA18 clones (i.e., cluster II versus clusters IV/V). Of the sSNVs with statistically significant restriction to fewer cortical regions than expected, the domain of BA18>BA17 sSNVs appeared more anterior to that of BA17>BA18 sSNVs (Figure 3Bii). Two BA18>BA17 sSNVs (chr17:10286416 and chr4:140684064) present at low MFs in BA18 were detected at ultra-low MFs in frontal BAs (BA4 and BA6, Figure 3Bii; Table S2.1). On the other hand, one BA17>BA18 sSNV (chr12:21352176) found in significantly fewer regions than expected for its MF (*q* < 0.01) was found at ultra-low MFs across the temporal and parietal cortex but at regions more posterior to the boundary of BA18>BA17 variants (BA37, BA41/42, BA7, and BA31; Figure 3Aiii). Additionally, this specific sSNV was not detected in the frontal lobe, unlike BA18>BA17 sSNVs at similarly low MFs. Second, MIPP-seq further showed how low- and ultra-low-mosaic sSNVs in the visual cortex may still cross over into immediately neighboring regions. Two variants in UMB4638 (chrX:86680485 and chr2:226043457) were discovered in BA17 at ultra-low and low MFs (respectively) but were also detected in BA18 at ultra-low MFs (Figure 3Biii). These data strongly suggest intermingling of occipital lobe lineages even late in cortical development. Finally, MIPP-seq suggests that even low-mosaic, late-rising BA9 variants may disperse into neighboring and distant territories but not as far as the occipital cortex, suggesting general restriction to the anterior cortex. For example, chr9:26385808 in UMB4638 (Figure 3Bi; Table S2.1) was detected in two adjacent cortical areas: BA9 (ultra-low mosaic) and BA10 (low mosaic). Similarly, chr2:230199483 in UMB4643 is detected throughout the frontal cortex beyond BA9 (Figure 3Ai; Table S2.2). Two other BA9>BA17/BA18 sSNVs in UMB4638 (chr2:240009733 and chr2:59535794), present at ultra-low and low MFs (respectively) in BA9, were detected as far away as the temporal lobe (BA20–BA22) at low MFs (Figure 3Bi).

In summary, lineage trees, coalescent models, and regional sequencing suggest that the asymmetric clonal distribution across the BA17/BA18 border may arise from two different developmental paths for early cortical progenitors (Figure 4D). First, progenitors may be restricted to BA9 (in the frontal cortex) between PMW2 and PMW9, with a possible transition through BA18 that leaves behind BA18-restricted descendant neurons by GW16 (GABN/GABNI and III). Second, progenitors may be restricted to the visual cortex and distribute across the BA17/BA18 border (IV and V).

### Combined sSNV and single-nucleus RNA-seq analysis suggests a late common cortical progenitor for glutamatergic and some GABNs

**Figure 5.**
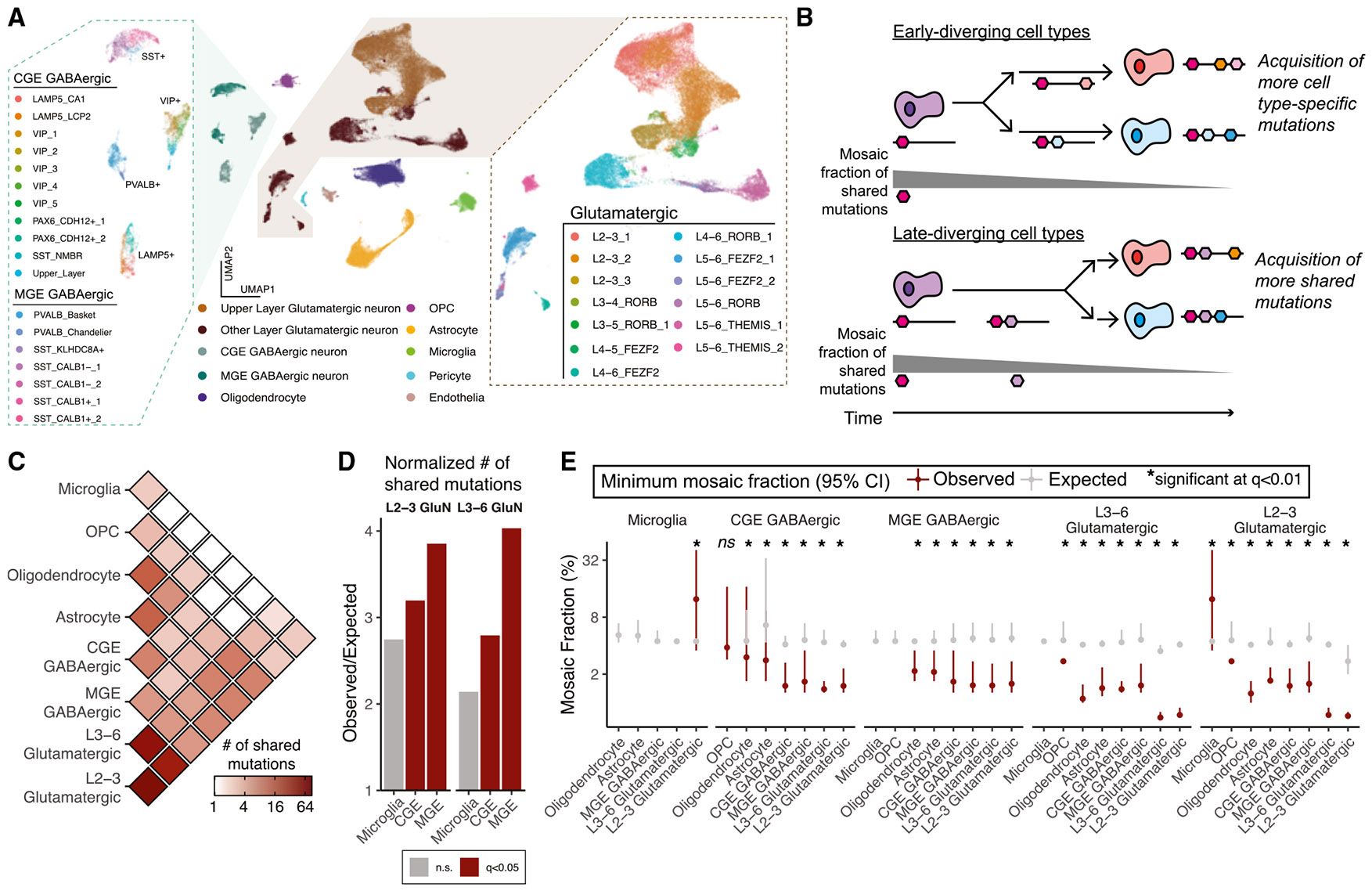
Co-occurrence of low-mosaic clonal sSNVs in glutamatergic neurons and GABAergic interneurons (A) Uniform manifold approximation and projection (UMAP) of snRNA-seq data (71,461 nuclei) taken from 2 donors (UMB4638 and UMB4643) across three regions (BA17, BA18, and BA9). GABAergic neuron (GABN) and glutamatergic neuron (GluN) subtypes are expanded in the insets. (B) A schematic prediction for the numbers and mosaic fractions of shared variants appearing within cell types that diverge from a progenitor cell type early (top) or late (bottom) in time. (C) The number of sSNVs shared between pairs of cell types. (D) Ratio of the observed versus expected normalized numbers of sSNVs shared between GluNs and microglia or different GABN subsets (see STAR Methods for details of calculation). (E) The minimum mosaic fractions of sSNVs (with 95% CIs) shared between major cell types and either microglia (far left) or neuron subtypes (CGE or MGE GABNs, L3–L6 or L2–L3 GluNs). Observed and expected variants correspond to those in (D) (see STAR Methods for details). Measurements and statistical tests for cell-type pairings with insufficient shared sSNVs or supporting cells are not shown.

Although single-nucleus RNA sequencing (snRNA-seq) yields sparse coverage for sSNVs,^31,50^ the presence of pre-specified variants can be verified in a fraction of cells, allowing us to study cell-type-specific somatic mosaicism. Thus, we collected snRNA-seq data from 10× Chromium libraries of DAPI-sorted or NeuN+-enriched fluorescence-activated cell-sorted (FACS) cells from BA17, BA18, and BA9 in UMB4638 and UMB4643. After extensive quality control, we retained 71,461 nuclei across 13 snRNA-seq experiments encompassing 37 different cell types based on the Allen Brain Atlas^51^ (STAR Methods; Figures 5A and S5A; Table S4.4). We profiled 350 candidate sSNVs in UMB4638 and 306 in UMB4643, including those used for lineage tracing and additional variants subjected to amplicon validation (STAR Methods; Table S4.3). Across all 15 experiments, although we obtained on average 1–4 unique molecular identifiers (UMIs) per site per cell type, we observed a wide overall distribution of UMIs per site over all cell types (1–4,096; Figures S5B-S5D; Tables S4.1 and S4.4). To increase power for quantitative analysis of sSNVs in cell types, we grouped the 18 GABN subtypes into CGE and MGE (caudal and medial ganglionic eminence, respectively) GABNs and the GluN subtypes into “upper layer” (the 3 L2–L3 subtypes) and “other layer” (comprising middle- and deep-layer GluNs).

To quantitate cell-type relatedness, we analyzed sSNVs (sample matched, or “observed”) shared across cell types, reasoning that the MFs of sSNVs (estimated from the WGS) shared in two cells will be higher if the cells diverged earlier in developmental time (Figure 5B). Assuming neutral selection, the last variant to have arisen in the MRCA of two cell types will have the lowest MF of all variants within this MRCA. Thus, we took the minimum MF (minMF) as our estimate for the MF of the last shared variant. We also reasoned that true sSNVs would reveal minMF estimates corresponding to basic cellular hierarchies. For example, the minMF of sSNVs shared between GluNs and microglia is much higher than that of sSNVs shared between GluNs and other GluNs, reflecting how the MRCA of microglia and GluNs existed during early embryonic divisions well before the brain-restricted MRCA of cortical GluN subclones. As a control, we used sSNVs from unrelated individuals (sample unmatched, or “expected”). Such sSNVs are often exclusive to a single individual, but experimental noise may generate their alleles at a low rate in sequencing data from a different individual, so unmatched sSNVs would yield consistent minMFs regardless of cell-type pair. Thus, we used sSNVs from 72 individuals previously profiled using 250× WGS.^38^ The lack of correlation between the MFs of sample-unmatched sSNVs in snRNA-seq with WGS, as opposed to the more visible correlation from sample-matched sSNVs, justified our control (Figures S6A and S6B). For further analysis, we required that at least 2 cells from each type support sSNVs (Figure S7B) and at least 3 sSNVs be shared to estimate the minMF (STAR Methods). We can robustly estimate minMFs down to ~50% of our snRNA-seq coverage (Figures S7A).

Strikingly, we observed several low-mosaic sSNVs found in both GluN and GABN subtypes (Figures 5C; Table S4.5). After normalizing by the number of mutated cells, the estimated number of shared sample-matched GluN/GABN sSNVs was significantly greater than the number of sample-unmatched variants (*q* < 0.05; STAR Methods; Figures 5D and S6C). Non-mutant and mutant UMIs were expressed for each variant in both neuronal subtypes, suggesting sufficient coverage and sensitivity (Figure S6D). From 32 sSNVs defining mixed GluN and GABN (GluN/GABN) clones at a ≤8% MF, we found that some GluN/GABN clones are regionally restricted (as some GluNs have been thought to be), while others are broadly dispersed across the cortex (as some GABNs have been thought to be) (Figure S6E). The snRNA-based MFs of GluN/GABN sSNVs were modestly correlated with their WGS AAFs (Pearson’s correlation coefficient of 0.35 for mutations with a <50% snRNA-seq MF; Figure S6F).

GluNs and GABNs have previously been reported to arise from anatomically distinct progenitors in rodents, with GABNs migrating into the cortex after arising from the MGE and CGE, the ventral telencephalic structures deep in the developing brain, separately from the dorsal cortical progenitors of the GluNs.^52,53^ However, several reports suggested an additional dorsal source of some GABNs in the human neocortex.^54-63^ Viral lineage tracing of human progenitor cells xenografted into the rodent cortex suggested that cortical ventricular zone progenitors produce proportions of 67%–85% GluNs to 4%–11% GABNs.^35^ If some GABNs and GluNs share a direct dorsal progenitor, then these neurons would likely share sSNVs present at lower minMFs. Moreover, we may expect some of these dorsally derived GABNs to show similar regional restriction patterns as their GluN siblings. Thus, we tested two signs of a dorsal progenitor: whether GluNs and GABNs consistently share low-mosaic sSNVs and whether these sSNVs show regional restriction or asymmetry in their associated neurons.

**Figure 6.**
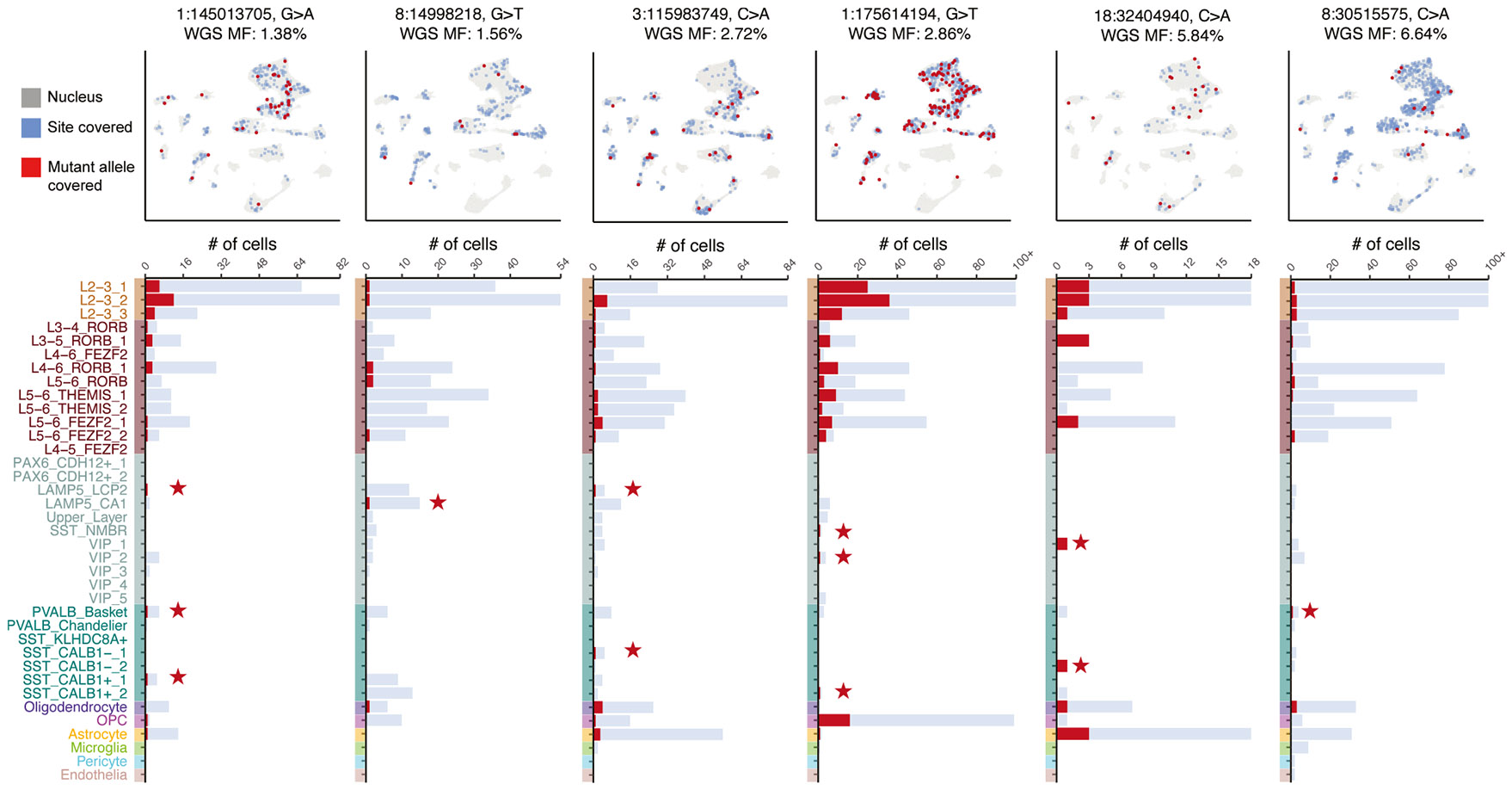
Low-mosaic sSNVs detected in both glutamatergic and GABAergic neurons Top: UMAPs depicting the cells carrying select sSNVs shared among GluNs and GABNs. Bottom: bar plots breaking down the coverage at the sSNV site per cell subtype. Numbers of reference- or alt-UMIs and alt-UMIs only are colored as in the UMAPs. Subtypes of GluNs and GABNs are colored by the original broad annotation as indicated in Figure 5A.

We were surprised to find low minMFs (<2%) shared not only within GluNs (GluN/GluN, <1% MF) and GABNs (GABN/GABN, <2% MF) but also between GluNs and GABNs (GluN/GABN) (Figure 5E; Table S4.6). minMFs of sample-matched sSNVs showed significant variation across different cell-type pairs, as opposed to sample-unmatched sSNVs (Figure S7C). For most cell-type pairs, sample-matched sSNV minMFs were statistically significantly different than expected (Figure 5E; *q* < 0.01, estimated from two-sided *t* test *p* values) and mirrored the progression from cell types not of cortical origin (microglia, minMFs ~10%) to layer-specific GluNs (minMFs < 1%). The minMFs of GluN/GABN sSNVs were comparable to those of sSNVs shared among different GABN subsets and sSNVs shared between neurons and macroglia (astrocytes and oligodendrocytes). As macroglia may arise from the same radial glial progenitors as neurons,^64,65^ the minMFs suggest a common ancestor of GluN/GABN clones present at the same time as neurons and macroglia. The low minMFs (<2% MF) between CGE-GABNs and GluNs support previous claims of a developmentally recent cortical ancestor of the two cell types.^35^ In several examples (Figure 6), we found that these low-mosaic GluN/GABN sSNVs showed up in upper-, middle-, and deep-layer GluNs and both CGE- and MGE-GABNs. The composition of clonally marked neurons suggests that diverse GABN subtypes might share late ancestry with GluNs in the human cortex and that these neurons can populate all layers of the cortex.

### Composition and regional restriction of low-mosaic clones comprising GluNs and GABNs

**Figure 7.**
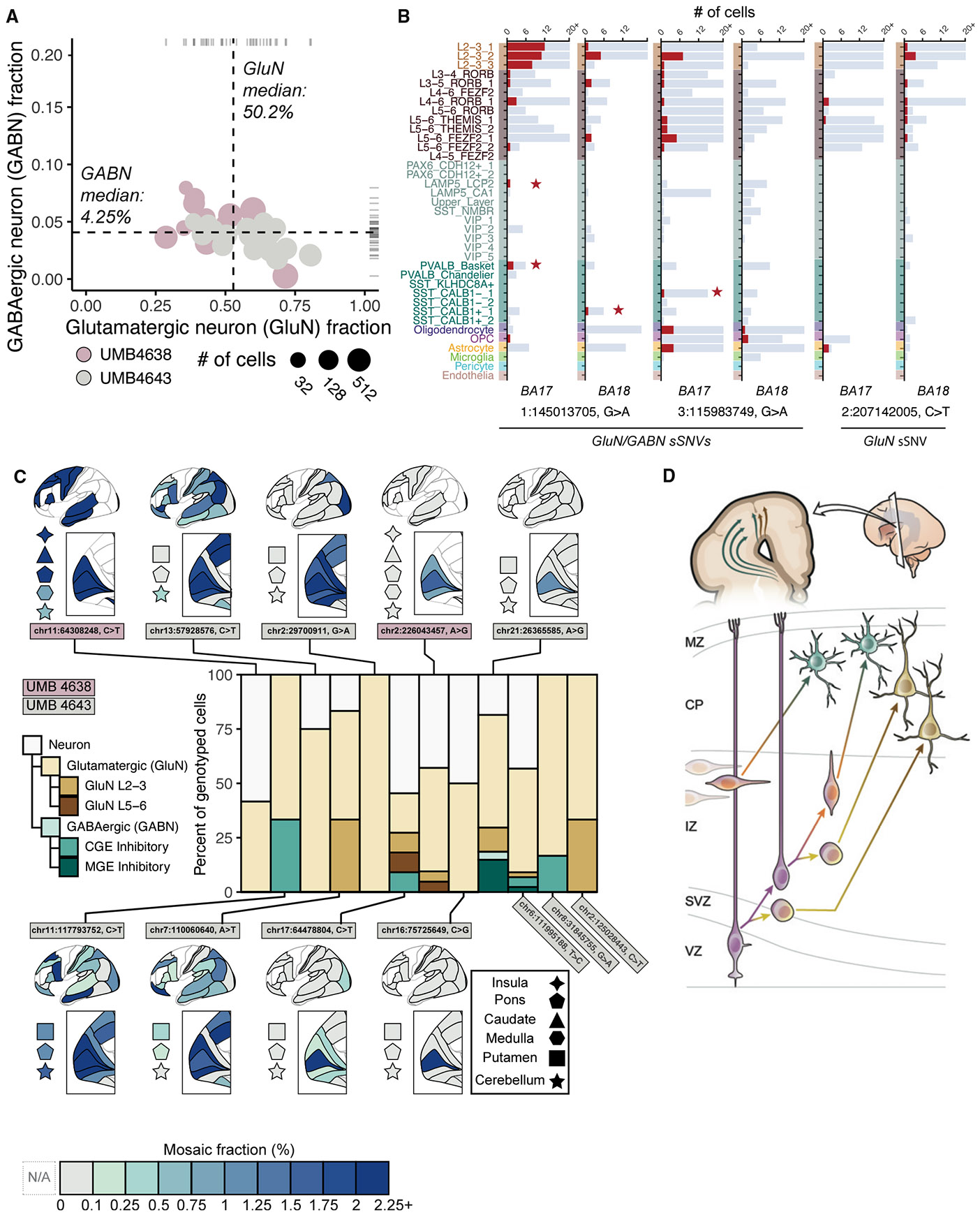
Composition and regional distribution of low-mosaic glutamatergic and GABAergic clones (A) Empirical Bayes estimates of the proportion of GluNs and GABNs found in somatic clones. Only clones with ≥10 cells were analyzed. (B) Examples of clones defined by low-mosaic sSNVs with either BA17- or BA18-restricted GluN presence. Plots are formatted as in Figure 6. (C) PRDD-seq data (co-capture of sSNVs from RNA and DNA in the same cell) confirming the co-occurrence of GABNs and GluNs in the same low-mosaic clones. MIPP-seq data showing regional distribution are available for 9/12 variants. (D) Schematic model of cortical co-generation of GABNs and GluNs; color scheme follows that of (C).

Having identified GluNs and GABNs sharing low-mosaic sSNVs, we sought to analyze their composition and regional restriction. From clones inferred by Louvain clustering on snRNA-seq (Figure S8A; Table S4.7; STAR Methods), empirical Bayes estimation yields GluN:GABN ratios of approximately 12:1 (GluN:GABN of 50.2%:4.25%; 95% CIs of 39.0%–69.1% and 4.14%–7.6%, respectively; Figures 7A and S8B), surprisingly similar to ratios seen from xenograft experiments.^35^ Next, we examined if some of the GluN/GABN clones show regional restriction or asymmetry across BA17 or BA18. Most did not show significant regional asymmetry, although smaller (<50 cells) clones did (Figure S8C). To investigate this further, we examined individual variants. For two of the sSNVs marking GluN/GABN clones (Figure 7B), we observed regional differences in the cell types of their constituent neurons. One of these sSNVs (1:145013705, G>A) was shared by GluNs and GABNs in both regions, whereas a second (3:115983749, G>A) was found in multiple cell types in BA17 but was restricted to glia in BA18. This latter sSNV appeared in our single-cell lineage tree and was predicted to have arisen at PMW10 (GW20), as did a third GluN/GABN sSNV (3:65583407, C>T) (Figure S9A). A fourth sSNV, overlapping our lineage tree (8:42336842, C>T), appeared only in L2–L4 GluNs (Figure S9B) but arose at PMW6 (GW16), later than the GluN/GABN sSNVs. Mirroring the observed GluN/GluN and GluN/GABN variants, we also located an sSNV (2:207142005, C>T) restricted to GluNs but showing significant skew toward BA18 rather than BA17 (Figure 7B), a sign of late clonal origin. Taken together, these findings suggest that GluN/GABN progenitors may follow consistent differentiation patterns, may arise late in neurogenesis, and show some evidence of regional restriction like GluN-restricted clones. The data suggest that the low-mosaic GluN/GABN clones represent a dorsal cortical progenitor, though this would require additional studies to confirm.

To further explore regional patterns in GluN/GABN clones, we used parallel RNA and DNA analysis after deep sequencing (PRDD-seq) for targeted analysis of multiple RNA markers of cell type and multiple sSNVs within the same single cells.^66^ We applied PRDD-seq to 14 sSNVs of UMB4638 and UMB4643 for which we could successfully design PRDD-seq primers. We could confidently isolate, genotype, and classify the cell types for neurons carrying 12 of these sSNVs, 9 of which had topological information from MIPP-seq.

Low-mosaic sSNVs showing diverse spatial patterns (widespread, non-regional, or lobe restricted; chr11:64308248, chr11:117793752, chr13:57928576, chr7:110060640, and chr2: 29700911) were commonly seen in GluNs but also included GABNs in several cases (Figure 7C; Table S5). These sSNVs confirm that clones dispersing across several cortical boundaries at low MFs represent GABNs, but the pattern reinforces our observation that some GluN clones may also disperse across cortical boundaries.

Several regionally restricted sSNVs centered around BA17 marked both GluNs and GABNs (Figure 7C; Table S5), further supporting a late shared dorsal origin. For example, chr21:26365585 (restricted to BA17) was found in 17 GluNs and 5 GABNs, with 5 other identified neurons not further assignable to subtype. chr17:64478804 (≤2.14% MF) was detected in all occipital lobe areas (BA17, BA18, and BA19) and in 11 neurons (4 GluNs, 1 GABNs, and 6 neurons not further classified; Figure 7C). Two of the PRDD-seq sSNVs tagged both CGE- and MGE-GABNs along with L2–L3 GluNs, further supporting the co-generation of GluNs and mixed GABN subtypes. Since GABNs are both less common and expected to be widely dispersed, our single-cell methods are relatively insensitive to sampling clonally related GABNs. Nonetheless, the consistent co-occurrence of GluN/GABN sSNVs based on two different technologies and the evidence for regional restriction of clonally related GABNs and GluNs both support the presence of a shared dorsal cortical GluN/GABN progenitor relatively late in neurogenesis (Figure 7D).

## DISCUSSION

Using somatic mutations as markers of cell lineage, we find major aspects of human cortical cell lineage that differ considerably from what has been described in animal models to this point. Deep WGS, single-cell lineage tracing, and MIPP-seq suggest that ultra-low-mosaic sSNVs may disperse widely across the cortex in general but show prominent non-uniformities across the BA17/18 border. BA17 harbors more regionally restricted sSNVs than BA18, likely reflecting regional differences in proliferation, and clonal intermingling appears somewhat restricted across this border. In addition, combining DNA analysis with single-cell transcriptomics suggests that widely dispersed clones contain many GluNs and reveal clones of GluNs/GABNs likely arising from the dorsal proliferative region *in vivo*.^60,62,67^

Our data are consistent with studies of non-human primates showing a sharp change in patterns of proliferation in the subcortical proliferative zones beneath the BA17/18 border, as well as relatively constrained patterns of radial migration of BA17 neurons.^41,43-45^ In single-cell tracing studies of the E78 subplate in macaques that underlies BA17/18, BA17 showed more radial trajectories of migrating supragranular neurons than BA18,^44^ in addition to a unique dependence on visual inputs for its proper development. The high neuronal density observed in BA17 compared to BA18^39,68^ may reflect the observed higher tendency for sSNVs identified in BA17 to be restricted to BA17, compared to BA18 or BA9, where sSNVs are not as commonly restricted to the region where they were discovered. To reconcile our data with these findings, we propose that in addition to dispersion patterns seen elsewhere in the cortex, clones at the BA17/18 border also show BA17-restricted increased proliferation and clonal expansion, likely involving predominantly upper-layer neurons given the low MF of the observed BA17-restricted sSNV. Increased local proliferation of BA17 neuronal precursors would seem sufficient to produce both higher neuronal density and increased clonality marked by sSNVs detectable at our MF threshold. Alternatively, our data are also consistent with a model in which the border unevenly allocates progenitors between BA17 and BA18 and restricts BA17-derived clones from crossing over into the rest of the cortex, while BA18-derived clones are not as restricted and thus end up as far as the frontal cortex.

Our findings confirm earlier reports of broad dispersion and intermingling of clonal progeny in the human frontal and lateral cortex^29-32,34,69,70^ but show, for the first time, that these widely dispersed clones include GluNs. While previous studies have shown that specific GluN clones intermingle within a single cortical column,^66^ we observe that GluN clones present in all cortical layers typically encompass most or all of the cortical surface. GluN clones disperse across multiple cortical areas at MFs as low as <1%, especially in the frontal lobe, although previous limited analyses have suggested that later clonal events can show more limited dispersion across cortex at even lower MFs.^29^ Although the last-generated sSNVs are hard to recover, we have also found that sSNVs at a <1% MF with topographic restriction are often limited to neurons in middle-to-upper cortical layers. Confirming this finding will require analyzing more samples, but the observed extreme level of clonal intermingling in the frontal lobe for even rare, late-rising GluNs has major consequences for models of clonal structure in humans.^15^ The wider dispersion of GluN-generating clones in the human frontal cortex contrasts with the more coherent clonal patterns reported in the rodent cortex,^12,18,71^ though limited reports in larger-brained mammals such as ferrets and non-human primates hint at wider clonal dispersion in these species as well.^71-74^ It is unclear for now whether the human reflects a scaled-up version of similar mechanisms in non-primates or shows newly evolved mechanisms. One implication of the widespread clonal dispersion in humans is that pathogenic clonal somatic mutations, such as in focal cortical dysplasia, may be scattered widely and beyond the borders of visible cortical lesions caused by these mutations.^75-77^

Three observations that we have made support the dorsal co-generation of GluNs and GABNs *in vivo*: low-mosaic clonal sSNVs shared in both subtypes, a consistent 12:1 ratio of GluN:GABN progeny agreeing with xenograft experiments,^35^ and regional asymmetries in clonal neuronal distribution. Per traditional models, dorsally derived GluNs radially disperse but remain mostly proximal to the progenitor’s position in the ventricular zone, while GE-derived GABNs migrate and disperse widely into the cortex, crossing multiple BA boundaries. Presumably, GABNs co-generated with GluNs from the same dorsal progenitor would remain as regionally restricted as GluNs, and our data support this hypothesis. Moreover, our data support the co-generation of both CGE- and MGE-type GABNs with GluNs, suggesting that GABN progeny of this hypothesized dorsal progenitor may be more diverse than previously reported.^33,36^ Our work validates this finding with a complementary approach using mutational analysis within unbiased single-nucleus transcriptomics.

Whereas GABNs appear exclusively derived from subcortical sites in the mouse,^22,78^ dorsal sites, in addition to well-established subcortical sources,^21,79^ appear to significantly contribute to GABNs within humans. In comparison to global GluN/GABN ratios of 7:3, 3:1, and 8.5:1.5 in humans, marmosets, and mice, respectively^80-82^ our 12:1 ratio suggests that a substantial minority of the cortical GABNs in humans are dorsally derived. Somatic mutational studies in non-human models may evaluate whether the dorsal GABN source is a primate-derived addition or whether mice selectively lost this dorsal source. Additionally, our data do not exclude a ventral progenitor migrating from the GEs into the cortex before producing GluN/GABN progeny. Although we have profiled over two dozen such low-mosaic GluN/GABN sSNVs across two separate individuals, more sophisticated and efficient methods are required to study dorsally derived GABNs.

### Limitations of the study

Small sample sizes constrain human lineage studies due to a lack of inexpensive, high-throughput methods to simultaneously analyze single-cell whole genomes and transcriptomes. The vastness of the human cortex also remains an obvious challenge for systematic description. Human studies require retrospective analysis of lineage, which does not allow direct determination of where neurons are formed (only their final locations) and limits validation of inferred developmental parameters. Deep WGS and single-cell WGS have low sensitivity to detect the exceedingly low MFs of mutations at the “late branches” of neurogenesis, which are crucial to examine regional and cell-type decisions. The lack of sensitivity especially impacts the analysis of GABN lineages, which, in animal models, are highly dispersed^26,60,62,83,84^ and which likely correspond to the outlier sSNVs that we observe with cortex-wide dispersion at very low MFs. New approaches such as duplex sequencing^85,86^ promise to improve sensitivity for late-occurring variants.

## STAR★METHODS

### EXPERIMENTAL MODEL AND STUDY PARTICIPANT DETAILS

Human tissues were obtained from the NIH NeuroBioBank at the University of Maryland Brain and Tissue Bank. Fresh-frozen postmortem tissues from two neurologically normal individuals were used in this study: UMB4638 (a 15-year-old female) and UMB4643 (a 42-year-old female). UMB4638 died from motor vehicular injuries and UMB4643 died from cardiovascular disease. Both individuals had no known neurological or psychological diagnoses at the time of death. Both individuals were obtained as part of previous studies in our lab.^30,31^ We also used fresh-frozen postmortem cortical tissues from two other neurologically normal individuals for WGS analysis who had no known neurological or psychological diagnoses at the time of death: UMB5575 (a 17-year-old female who died of compressional asphyxia) and UMB5580 (a 29-year-old male who died of acute pancreatitis). All tissue samples were prepared according to standardized protocols (https://www.medschool.umaryland.edu/btbank/Researchers/Tissues-Collected and https://www.medschool.umaryland.edu/btbank/Medical-Examiners-and-Pathologists/Minimum-Protocol) under the supervision of the NIH NeuroBioBank ethics guidelines. Brodmann area identification and sampling were completed by the NIH NeuroBioBank at the University of Maryland Brain and Tissue Bank.

### METHOD DETAILS

#### Processing of human tissues and DNA samples

Cortical samples were biopsied from the left hemisphere of all individuals. For initial variant calling, bulk samples were biopsied from the PFC and occipital lobe, specifically BA17 and BA18. Likewise, single cells for variant calling were isolated from PFC (UMB4638: coronal section 3; and UMB4643 coronal section 4). For downstream experiments, including validation experiments, related to these three areas, biopsies from BA9 (representing PFC), BA17, and BA18 were used.

For all four individuals’ brains, bulk DNA was extracted from tissues using the lysis buffer from the QIAamp DNA mini kit (Qiagen; Cat. 51304) with proteinase K digestion and RNase A treatment, followed by a phenol-chloroform extraction and alcohol precipitation.

Single nuclei were isolated by fluorescence-activated nuclear sorting (FANS) using an anti-NeuN antibody as a neuronal nuclei marker (Millipore, MAB377). Nuclei were lysed on ice in alkaline conditions, and whole-genome amplified using MDA, as previously described.^29-31,100^

#### Human sample preparation

We received tissue biopsies from two neurologically normal individuals, UMB4638 and UMB4643, from the NIH NeuroBioBank. These samples have been used in prior publications for some limited variant discovery, WGS, and clonal analysis.^30,31^ The left hemisphere of each brain was analyzed, with the right hemisphere having been prepared by the NIH NeuroBioBank for histological analysis and thus unavailable for DNA sampling. Because these brains represent shared resources, many cortical regions had already been extensively sampled, especially primary motor cortex, primary somatosensory cortex, hippocampus, and other regions, and thus were unavailable for our analysis. These unavailable regions are indicated on the cortical maps in areas with no color shading and outlined with gray dashed lines outlining the representative BA regions and their unavailability.

The left hemispheres of each sample were sectioned coronally at ≈1 cm, according to neuropathological conventions and standard operating procedures. Approximate coronal section thicknesses were measured for coronal sections available for sampling (Figures S1B and S1C). Samples were requested from all cerebral cortical BA regions available and identifiable, and from most if not all subcortical and non-cortical brain sites as well. Biopsies of cortical areas were cut by dieners at the NIH NeuroBioBank, using extensive photographic maps and atlases of the human brain, recording the position of the sample relative to gyral landmarks, and the section number. Assignment of samples to BA regions comes from this biopsy process. Furthermore, photographs of coronal sections and tissues were taken before and after dissection for lucida tracing of biopsy locations within the coronal sections. Tissue samples are stored at −80° C until sample preparation. Sample preparations for bulk DNA extraction are as previously described.^30,31^ Biological duplicates for BA9, BA18, and BA17 were isolated from the same tissue biopsy with the same protocol but separately prepared.

#### Whole-genome sequencing library preparation

Deep (210X) whole-genome sequencing (WGS) on bulk tissue DNA was prepared using the Illumina TruSeq PCR-free preparation kit for paired-end barcoded WGS libraries. Paired-end sequencing (150 bp x 2) was performed on an Illumina HiSeq X10 (UMB4638 and UMB4643) or NovaSeq6000 (UMB5575 and UMB5580) instrument (Psomagen, Inc., Rockville, MD).

As described previously,^31^ single neuronal nuclei were isolated using FANS with NeuN staining, a neuronal nuclei marker. Single neuronal sequencing was prepared by shearing 100 ng of DNA of each sample on a Covaris Ultra-Sonicator to yield ~350 bp fragments. Paired-end barcoded WGS libraries were prepared using the Illumina TruSeq Nano LT sample preparation kit and paired-end sequencing (150 bp x 2) was performed on an Illumina HiSeq X10 instrument. Library preparation and sequencing were completed at the New York Genome Center (New York, NY). Sequencing data of ten single prefrontal cortex neurons from each brain, which were selected based on low allelic and locus dropout rates, were made available from a previous study.^31^

#### WGS data processing

WGS reads generated from single neuronal sequencing were processed as previously described.^31^ WGS reads from bulk tissue sequencing were prepared, in brief, by mapping reads on to the human reference genome (GRCh37) by Burrows-Wheeler Aligner (BWA) with default parameters. Duplicate reads were marked with MarkDuplicate of Picard tools, and further post-processing was completed with local-realignment around indels and base-quality score recalibration using Genome Analysis Toolkit (GATK, version 3.5).^88,101^

#### Somatic SNV variant calling in bulk WGS

sSNVs in bulk and single cell DNA WGS was called using Mutect2 (bulk calling), MosaicForecast (single-cell and bulk calling), single-cell Mosaic Hunter, and a GATK-based triple-calling strategy (single-cell calling) as previously described.^30,37,66,89,102^ sSNV calling in bulk DNA WGS using Mutect2 (version nightly-2016-04-25-g7a7b7cd) utilized the panel of normal tissue approach to identify candidate sSNVs, and we also completed a tissue-region versus tissue-region comparison to increase sensitivity in detecting regional mutations. All calls (pass and non-pass) from Mutect2 were considered to increase sensitivity and minimize the loss of potentially rare sSNVs. All other sSNV detection parameters, along with the associated filter thresholds, for the other call-sets were as previously described.^29-31,37^ The full set of sSNVs derived from all calling methods were then filtered for somatic mutations unique to each individual by excluding variants shared between both individuals. Variants located in segmental duplications and repetitive regions were also filtered out before designing the amplicon panel for targeted re-genotyping. We used MosaicForecast^37^ to identify candidate mosaic mutations from this final set.

#### Filtering T>Gs from UMB5575 and UMB5580

The brains of UMB5575 and UMB5580 were sequenced on a NovaSeq6000 as described above, and after our mutation calling workflows (Mutect2 on bulk WGS using the panel-of-normals, filtering out segmental duplications and clustered repeats, and running MosaicForecast), we observed a significant excess of T>G sSNVs. UMB5575 and UMB5580 were neurotypical postmortem donors who lacked Polη mutations typically associated with T>G hypermutation (especially in immunoglobulin genes, at which UMB5575/5580 did not show clustered mutations)^103,104^ and had no history of 5-fluorouracil exposure through chemotherapy observed to introduce T>G’s.^105^ Thus, we ruled out biological or clinical causes for the T>G/A>C excess. However, an initial report suggests that samples sequenced on a NovaSeq6000 may contain an excess of low-AAF T>G/A>C mismatches compared to HiSeqX10 counterparts, with these mutations subsequently appearing in the final VCFs of sSNV callsets when using the GATK/Mutect2 Best Practices.^106^ Separately, we have determined that an excess of 2–5% AAF T>G/A>C SBS’s is prevalent across multiple independent NovaSeq6000 sequencing datasets generated by independent research groups in different years,^107^ suggesting a recurrent sequencing artifact.

We determined that the T>G/A>C mutations were overenriched at TTCC/GA-repeat sequences. To identify and filter mutations at this motif in an unbiased fashion, we constructed a position-weighted matrix (PWM) from the 41-base window encompassing each type of single-base substitution (SBS) from UMB5575/5580 as a “test” and from UMB4638/4643 as a “control.” Then, for each SBS and each PWM, we computed a joint multinomial likelihood that all bases in a 9-base window centered on the mutation arise from the PWM and determined the likelihood ratio (of arising from the UMB5575/5580 PWM versus the UMB4638/4643 PWM). We empirically computed *p* values for each mutation; mutations at *p* < 0.05 (i.e., reject the null hypothesis that the sSNV is as likely to have arisen from the sequencing processes of HiSeqX10 as NovaSeq6000) were deemed artifactual and filtered out from downstream analysis.

#### Estimating the ratios of regional sSNV counts

Comparisons of the number of mosaic variants found in one brain region must consider several technical factors. Mosaic variant discovery pipelines can exhibit different sensitivities based on the AAF of desired variants; for example, the sensitivity to detect mosaic variants at AAFs of ≤5% is significantly lower than for variants at AAFs in the 5–30% AAF range. Higher-AAF variants can be shared across multiple brain regions, and brains can vary in the total number of variants discovered due to batch effects or sequencing platforms. Thus, we sought to estimate the ratio of variants present in BA17 versus BA8 (and similarly for BA9 versus BA17 or BA18), as a ratio would summarize a fundamental difference in the variants found in one region versus another due to one region having more region-specific variants or having variants at significantly higher AAFs. If two regions share a similar number and set of variants, then we assume that a variant detected in one of those regions would be found at a similar AAF in the other region. This latter case serves as our null model against which we can use to test whether the estimated ratio of variants in one region over another is significant.

In each of seven different AAF bins (1–2%, 2–3%, 3–4%, 4–5%, 5–10%, 10–20%, and 20–50%), we simulated the minimum number of sequencing reads at which we would detect a variant in the bin based on the AAFs of all variants found in this bin (our “threshold”). For each variant in each region, we simulated the number of sequencing reads at which it would be detected in that region as a binomial random variable with N equal to 250 reads and *p* equal to the AAF at which the variant is found in the region, and we retained the variant if the simulated number of reads exceeds that of our threshold. We estimated the projected number of similar variants that can be detected at the observed variant’s AAF by computing the reciprocal of the sensitivity estimated from a smoothed spline fitted to sensitivity estimates previously published for MosaicForecast.^31^ To obtain the ratio of projected variants in that region, we summed up the numbers of projected variants; this sum is used to compute the ratio of variants that exist in one region versus another. The ratio under the null model is estimated with a similar procedure, except a particular variant’s AAF in a region is simply the mean of the AAFs across all tested regions. For example, a variant detected at AAFs of 2% in BA17 and 1% in BA18 in the same brain would be simulated at an AAF of 1.5% for the control. Simulations were conducted over 1000 iterations, and the 99% confidence interval was computed to provide an interval estimate of the ratio.

#### Targeted amplicon sequencing of bulk DNA

In UMB4638 and UMB4643, we validated identified mutations using deep amplicon sequencing in 37 brain samples and 18 non-brain tissues samples (Table S1.1). Adrenal tissue was not specified whether the sampled was biopsied from adrenal medulla (derived from ectoderm) or adrenal cortex (derived from mesoderm), so it is listed as mesoderm-ectoderm (Table S1.1). Candidate sSNVs were selected based on parameters set by the amplicon design pipeline requiring primers mapping to unique genomic regions. Targeted regions were captured by the amplicon panel in bulk unamplified DNA samples from both brain and non-brain tissues (Table S1.1). Targeted sequencing of bulk DNA samples was completed using a custom designed amplicon pool and a custom library preparation and barcoding protocol. A custom amplicon panel for each individual was designed to target specific candidate sites using the Ion AmpliSeq Designer tool (Thermo Fisher Scientific). Each amplicon pair was designed to be unique and specific to a target candidate site. The amplicon panel was used in the initial targeted capture step with minimal PCR cycles to reduce artifacts from PCR amplification. The initial input amount of DNA was 20 ng per reaction, consisting of 9 μL of 2X custom AmpliSeq Primer Pool and 10 μL of 2X Phusion U Mastermix (Thermo Fisher Scientific, F-562). Targeted amplicon sequencing of bulk tissue DNA was prepared using a custom library prep protocol for paired-end barcoded WGS libraries. Paired-end sequencing (150 bp x 2) was performed on an Illumina HiSeq X instrument. Library sequencing was completed by Psomagen, Inc. (Rockville, MD).

For all targeted captures using the custom amplicon panel, two biological duplicate bulk DNA samples representing two separate extractions from the same tissue region were used. For technical replicates, each biological duplicate was prepared three times, for a maximum of 6 samples for each evaluated tissue. For controls, preparations were also performed using 1) nuclease-free water, 2) an unrelated male fibroblast genomic DNA sample (Promega, G1471), and 3) DNA from the other individual (i.e., using the custom panel specific to UMB4643 on UMB4638 bulk DNA).

Specifically, regarding the region-validation experiments, the following samples for BA17 and BA18 were used: bulk tissue DNA samples used for the original sSNV detection, and a biological duplicate sample extracted similarly from the same tissue but not used for candidate sSNV discovery. For PFC, two biological duplicate samples were extracted similarly from within the same BA9 region; this exact BA9 tissue biopsy was not used for the original SNV detection. Custom amplicon panels were also used to target sSNVs in additional bulk DNA samples extracted from both brain and non-brain tissues.

Sequencing reads were prepared by first trimming the reads for quality and removing any leftover adapter sequences from the reads using CutAdapt^90^ (-q 20, -u −5, -U −5, -a AGATCGGAAGAGC -A AGATCGGAAGAGC). Next, common sequencing artifacts were corrected using the Pollux software^93^ to generate both error-corrected and original fastq files using the following settings: -p -n true -d true -h true -s false -f false. Reads were then mapped onto the human reference genome (GRCh37) by BWA-mem^87^ with default parameters. Further post-processing was completed with local-realignment around indels using Genome Analysis Toolkit^88^ (GATK, version 3.7, -T IndelRealigner –filter_bases_not_stored -greedy 1200 -maxReads 2000000 -maxInMemory 1500000), using all InDels from gnomAD version 1^108^ as a control set. Finally, all primer binding sites were clipped from the sequencing reads using Bamclipper^94^ and a bed file of all primers. Variants located within each amplicon were called using samtools mpileup version 1.3.1 (–output-tags INFO/AD,DP,AD -Q 20 -q 20).^91^ SNV variants were called alternate or reference using samtools mpileup.^91^ Finally, VCFs for each sample were processed to include 50 nucleotides flanking each side of the targeted mutation for estimating the background error rates.

All variant calls were further validated to distinguish true positives (TPs) from false positives (FPs) and germline events using a combination of public databases–gnomAD version 3 (v3)^108^–manual review of genome mappability, control tissue sequencing, and the comparison of the original tissue in which a mutation was identified against other tissues in the individual. True positive mutations were defined as being high quality sites with good mapping, rare/absent in gnomADv3, absent in control DNA samples, AAF of 0.5%–35%, and an allele depth (AD) > 2. Furthermore, the high confidence mosaic alleles were required to be detected within the tissues where they were originally identified. However, given some variability in amplicon sequencing depths, mutations not detected in the original tissue can also be considered as valid mutations given that they meet all other criteria and are present in multiple library preparations. Mutations additionally identified with an AAF suggestive of a germline event in control DNA samples (Promega genomic control and unrelated individual’s brain tissue), that appear as common high-quality germline events in gnomADv3 with good read mapping, were flagged as true germline events. Alleles present in the control tissues, regardless of AAF, that are present in gnomAD version 3, with poor quality flags and poor read mapping were further manually curated to confirm their FP status. Finally, any mutation consistently identified as a germline event across all tissues of a given individual, with an average AAF of 40–60% across all samples, were manually reviewed and classified as a true germline event.

For validating of mutations called from UMB5575 and UMB5580, amplicons were submitted to Azenta Life Sciences for library preparation and sequencing using the Amplicon EZ protocol.

#### Lucida tracings and brain map annotations

Lucida tracings of sampled cortical sections (Figures S1B and S1C) were traced from photographs taken by the NIH NeuroBioBank at the time of tissue biopsy. Dashed lines indicate regions that are not present in photographs due to sampling prior to this study. Anatomy was extrapolated from records of sample locations, adjacent sections, photographs of right hemisphere formalin-fixed coronal sections, and atlases and MRI records of neurologically normal brain anatomy.

Lateral and medial cortical brain maps with Brodmann area (BA) annotations were adapted from the Brodmann (1909) areas (annotated) scene files for the left cortical hemisphere from the Brain Analysis Library of Spatial maps and Atlases (BALSA) database.^109^ Areas that are filled with color represent the corresponding MF of the sSNV in that BA sample.

#### Mosaic characterization using MIPP-seq

##### Capture of target sites using MIPP-seq

Mosaicism estimation of bulk tissue DNA was obtained using deep-targeted sequencing of regions captured by custom-designed primers, as described in a recently published method.^110^ When possible, ≥1 unique primers (termed “replicate primer pairs”) were designed to an SNV, with each additional replicate primer designed to stagger around the site of interest; this is to account for potential allelic dropout and imbalance, and to provide a more accurate mosaicism estimation of the targeted SNV. Every primer pair was designed with a sequencing adapter and unique barcode. Each primer pair was individually evaluated for a single expected product of correct fragment size and checked for efficiency. Custom-made multiplexed primer pools were generated and checked for primer cross-reactivity and capture efficiency. In brief, primer pairs were evaluated both independently and in pools, which were compared on a Tapestation D1000 ScreenTape system to check for proper product sizes. Replicate primer pairs targeting the same sites were placed in separate pools or used individually. Primer pairs that showed cross-reactivity with other primer pairs within a pool, such as abnormal fragment sizes, were isolated and ran in individual reactions as previously described.^110^ The targeted sequencing was prepared by running a PCR with the primer pairs and 50 ng of bulk DNA input on low cycle number (20 cycles). Libraries were prepared, and sequencing was performed on the Ion Torrent S5 sequencing platform. A calculation using an estimated 6–7 pg of DNA content per cell approximate ≈7,142-8,333 cells.^111^ Using this estimation, sSNVs with 0.1% MF, the lower limit of detection by this method, would represent ≈7–8 cells in the cellular population carrying the heterozygous sSNV.

##### MIPP-seq data processing and analysis

Raw unmapped BAMs consisting of uniquely indexed amplicon sequences were converted to fastq using “bedtools bamtofastq”^92^ prior to being demultiplexed into amplicon-specific fastq files based on their unique 15 nt barcodes with FASTX toolkit’s fastx_barcode_splitter (–bol –mismatches 3). Error correction was performed using Pollux^93^ (-n false -d false -h true -s false -f false), followed by barcode and quality trimming with CutAdapt (-u 10 -q 10). Each amplicon specific fastq was independently mapped against the human reference genome, hg19, using default settings in BWA-mem. Local realignment was performed using GATK version 3.7 IndelRealigner (-greedy 1200 -maxReads 2000000 -maxInMemory 1500000) using high quality indels extracted from the gnomAD genomes database. Finally, primer binding sites were clipped using the bamclipper tool^94^ with default settings.

Each locus site in each amplicon was evaluated as carrying the alternate allele if it met the following criteria: 1) a minimum of 10,000 reads at the site of interest; 2) carrying the primary alternate allele called during initial variant discovery; and 3) the mosaicism at the given site is ≥ 0.1% MF (0.05% AAF). AAF averages were reported for those SNV sites with multiple (replicate) primer pairs. As previously described, the lower detection limit of mosaicism estimation using 50 ng of DNA input is 0.1% mosaicism (0.05% AAF).^30^ For the graphical presentation of mosaicism on the brain map figures, any sites with <0.1% mosaicism or carrying the reference allele, but passed the minimum total read limit (10,000 reads per site), were categorized as “alternate allele absent” for that tissue (represented as the shaded gray areas on the brain maps). If a given site yielded <10,000 reads at the site of interest, it was designated as inconclusive (represented as non-shaded areas with gray dashed lines on the brain maps).

Background error rates (Figure S2A) were calculated as previously described.^30^ In brief, background error rates of mutations were calculated using the average allelic fractions within 100 bases surrounding the targeted SNV in each amplicon. This represents the likelihood of generating a mutational artifact. If multiple (replicate) primer pairs were designed to the target site, then an average background error rate was calculated for that specific SNV across the relevant primer pairs.

All sSNVs followed for subsequent spatial mosaic analysis had background error rates below the lower technical limit for signal detection (0.1% MF), indicating the level of sensitivity provided by MIPP-seq (Tables S2.3 and S2.4). A comparison of all sSNVs in biological duplicates of the same cortical areas shows that most sSNVs have similar MF values across biological replicates (Tables S2.5 and S2.6; Figure S2B). In all, 32 sSNVs and 27 sSNVs were studied for UMB4638 and UMB4643, respectively.

##### Simulated null model for MIPP-seq analysis

We sought to generate a null model of the number of regions in which we would expect to find a sSNV from MIPP-seq. Under this null model, spatial structure in the cortex does not affect sSNV distribution across regions, so sSNVs are uniformly mixed in each region such that the MF of the sSNV in one region is close to the cortex-wide average MF of the sSNV. We averaged the sSNVs’ MFs from WGS to generate this cortex-wide average. We also averaged the cortex-wide error rate of a sSNV’s MIPP-seq measurements. We simulated MIPP-seq experiments in which we binomially sampled the number of mutant reads from 10,000 theoretical reads using the WGS-inferred average MF as the probability parameter. We also simulated the number of error reads using the average error rate. A sSNV was said to be “detected” in a region if it appeared in more simulated mutant reads than error reads and if the fraction of mutant reads exceeded 0.1%. We conducted 1000 replicate simulation for as many regions as were tested for each individual sSNV. The expected range is simply the minimum and maximum number of regions from the 1000 replicates.

#### Grouping of SNVs into somatic and germline categories

SNVs were grouped into somatic (ultra-low mosaic, low mosaic, and higher mosaic) or germline categories based on the average mosaicism (2 x alternate allele fraction (AAF) percent of an SNV across all evaluated samples. Grouping was also further confirmed by the mutation categorization completed by targeted amplicon sequencing (see Table S1.6). SNVs with an average AAF of ≥45%–50% for a heterozygous SNV were grouped as germline mutations. Somatic mutations were categorized as higher mosaic sSNVs if the average mosaicism was between 10 and 90% MF. Low mosaic sSNVs carry a mosaicism of 2–10%. This category range is based on the lower limit of detection (10% MF) for standard sequencing technologies for mosaic mutations, including Sanger sequencing, pyrosequencing, and standard exome sequencing. Ultra-low mosaic sSNVs are sSNVs with an average mosaicism of ≤2% across all evaluated tissues. Our previous work demonstrated the appearance of general restrictions within the cortex beginning at 4.3% mosaicism for heterozygous SNVs isolated from BA9, with mutations at >5% mosaicism appearing widely outside the brain.^31^ An additional study evaluating early human development using early-occurring sSNVs showed that brain-specific progenitors produced clones with average MFs of <2.5% across the cortex in one individual.^41^

#### Analysis of panel single-cell MDA data

We used multiple displacement amplification (MDA) to capture 122 brain-restricted sSNVs (56 for UMB4638 and 66 for UMB4643) across 1131 single nucleus genomes (563 in UMB4638 and 568 in UMB4643) taken from BA17, BA18, and BA9. We used cutadapt^90^ with error rate set to 50% to aggressively trim adapters, partial adapter sequences, poly-G sequences, and polyX-sequences from demultiplexed FASTQ files of the panel single-cell MDA (pscMDA) experiment. We aligned all reads to hg19 (GRCh37) using bwa-mem. We genotyped each mutation in our panel from the FASTQ data using procedures described before.^31^ Briefly, the genotyping model assumes that the posterior probability of a site being somatic-mutant in a cell can be computed from a binomial likelihood of observing alternative-allele backing reads at observed counts at probability *p*, i.e., the expected read fraction of a somatic-alt variant in the cell (ideally at 0.5 but potentially different given allele imbalances introduced during amplification). The posterior probability of a site being non-mutant at a site is also computed from a binomial likelihood of observing erroneous (i.e., non-reference) reads at the site. The prior probability of a site’s genotype within a given cell is proportional to the observed read fraction of the mutant allele across all cells.

All parameters are estimated from heterozygous SNPs introduced in the panel and off-target amplifications that serve as examples of reference-homozygous sites. For instance, amplifications of UMB4643 sites in UMB4638 samples were used to estimate parameters for the reference-homozygous genotype in UMB4638. The two batches of sequencing data for the same cells and sites were genotyped separately before generating a consensus genotype matrix, using the estimated mosaic (cell) fraction of the variant to compute a binomial probability of the variant being somatic-mutant within a given cell across both batches. All parameter fittings were conducted using JAGS implemented through R, and code to genotype cells and sites is provided on the repository linked under the linked repository. We genotyped >85% of the sites across 1124 nuclei (Figure S4C), with mosaic fractions of each variant correlated with the MFs estimated from the AAFs measured by deep targeted sequencing of these variants (Figure S4B).

#### Single-cell lineages and coalescent model

We assume that mutations evolve neutrally within the lineage (i.e., negligible chance of recurrent mutations newly arising on separate branches of a lineage tree without being inherited from a common ancestor). We applied scistree^95^ to our consensus genotype matrix to filter out genotypes with this model (for example, for two sites [A,B], subpopulations exist with genotypes [0,1], [1,0], and [1,1]) and impute compatible genotypes constrained by variants’ mosaic fractions. We used mpboot^96^ to construct maximum parsimony trees from this imputed genotype matrix. Genotype matrices from before and after imputation are reported in Figure S4A. Full mathematical details of the coalescent model are provided in Data S1.

#### Calculating regional restriction statistics

We inferred coalescent timings and constructed lineages agnostic to the regions where mutations were detected. To assess each clade’s association with different regions, we constructed the regional restriction statistic (RRS), which we formalized as the logodds ratio of the distance between two cells in a clade belonging to the same region to the distance between cells from different regions. We envisioned this ratio as describing whether two cells from a particular clade and from the same region tend to be more closely related than if those cells were from different regions. For each pair of cells within each clade, we computed the ratio of the phylogenetic distance between cells within the same region to the distance between cells from different regions, and we estimated the mean and standard deviation of the distance ratios for each clade. The mean ratio is the RRS for a clade.

An RRS close to 1 suggests that cells within this clade are equally related whether in the same region or in different regions, suggesting that the variant’s dispersal throughout the cortex is not significantly shaped by or associated with regional separation. An RRS significantly above 1 suggests that cells in this clade are more related to clade-mates within the same region than across regions, suggesting that the clade is restricted mostly to one region or asymmetrically allocated to one region. An RRS significantly below 1 suggests that clades are more related to clade-mates from different regions than within the same region, suggesting that the clade widely populates other regions early on prior to the occurrence of later-stage variants. A null RRS can be defined using early-mosaic or germline mutations, which should precede the formation of brain regions and subsequent allocation of cells among them.

#### Single nuclei RNA-seq processing and analysis

Single nuclei and bulk 10X Chromium Genomics^112^ gene expression datasets were prepared from three different cortical areas from UMB4638 and UMB4643. 10X single nuclei RNA sequencing data was generated from sorted cells using either DAPI or NeuN (neuronal nuclei marker) from the BA17 and BA18 areas from both individuals. Previously published DAPI-sorted snRNA-seq libraries from BA9 were also analyzed from both individuals.^31^ We used CellRanger v7.0.1 to produce alignments (to an hg19 ENSEMBL version 17 reference) and raw count matrices, as CellRanger v7+ produces both exon and intron counts for genes in the raw UMI counts matrix. We applied CellBender v0.3.0^99^ to remove background noise (such as empty droplets and ambient RNA). We also computed intron fractions of transcripts within individual droplets using DropletQC^97^ to identify those further contaminated with cytosolic or ambient RNAs. Using both exon and intron read counts when producing the UMI counts matrix increases the count signal per gene and can improve the detection of cell types in our dataset, while the removal of empty droplets and ambient RNAs in snRNA-seq can help unmask rarer cell types by removing spurious clusters of nuclei or cytosolic transcripts from damaged cells that may contaminate the true transcriptional program within other nuclei.^113,114^ We produced Seurat objects for each of the 13 datasets using Seurat v5.0.1^98^, removed droplets with fewer than 200 UMIs and applied batch correction and dataset integration with Harmony.^115^ We conducted clustering after identifying variably expressed genes in the dataset. To filter out doublet droplets, we annotated Seurat-identified clusters with the expression levels of marker genes curated by the Allen Brain Atlas^51^ for basic brain cell types (GluN and GABNs, oligodendrocytes, OPCs, astrocytes, microglia, and endothelial/vascular cells). Clusters expressing high levels of mutually exclusive marker transcripts (e.g.,: >50% of cells in the cluster expressing neuronal and microglial markers) were deemed contaminated with doublets and removed. Clusters with median intronic fractions <0.5 were deemed contaminated with ambient RNAs and filtered out. We iterated between rounds of cluster evaluation/filtering, variable gene expression recalculation, and reclustering before we obtained a final set of clusters in which each expresses a set of marker genes from one of our broad cell types and each has a median intronic fraction of 0.6–0.8. Marker gene expression was used to annotate the remaining clusters with the major cell types. We also removed a small number (<40) of nuclei in our NeuN+-sorted libraries that were annotated as non-neuronal. To annotated GABN and GluN subtypes, we separately isolated clusters annotated as each broad cell type and conducted rounds of further variable gene identification, reclustering, and cluster evaluation/filtering to remove any lingering spurious droplets. We used label transfer to annotate the remaining nuclei with the layer-specific subtype annotations from the Allen Brain Atlas. After all quality control, major cell type identification, and neuronal subtype annotation; we retained 71,461 nuclei for downstream analysis.

#### Evaluating shared sSNVs counts and minMFs

For each pair of cell types in our dataset (in which GluNs were grouped into upper-layer and other-layer and GABNs were grouped into CGE and MGE), we counted the number of sSNVs in which the mutant allele was found shared by at least 2 cells from each cell type. When evaluating sSNVs shared by cells within a single cell type, we required that at least 2 distinct cells of the same type shared the mutation. The number of such sSNVs shared between cells of each pair of type was divided by the total number of pairs of cells that shared mutations overall, which is intended to control for the overall number of cells across a pair of cell types capturing mutations. The resulting number of sSNVs per mutant cell pair represents a normalized estimate of how many shared sSNVs are expected between two cell types.

We computed the normalized shared sSNV counts for both sample-matched (“Observed,” i.e., true mutations) and sample-unmatched (“Expected”) sets of sSNVs in the UMB4638 and UMB4643 snRNA-seq data. Two cells sharing an alternate allele at a sample-unmatched sSNV represent the cells sharing a spurious cell type relationship due to both experiencing experimental noise. For a given pair of cell types, we sought to evaluate if the normalized counts for true mutations are significantly greater than those of sample-unmatched sSNVs. We thus evaluated whether the difference in the two measurements is significantly different. We simulated a distribution of null differences from 10,000 pairs of bootstrap samples. Each sample was constructed by randomly sampling test and null normalized counts, and the difference in means between the samples was taken. From this simulated null distribution, we computed the upper-tail cumulative probability for each test sSNV to generate a *p* value, and at *p* < 0.05 we rejected the null hypothesis that two cell types do not share more mutant UMIs at a locus than non-reference UMIs created by experimental noise.

The minimum mosaic fraction (minMF) statistic was obtained by drawing bootstrap samples of mutations shared between two cell types, estimating the mosaic fraction from the observed AAFs of the mutations (MF is estimated as 2 times the AAF from WGS data where the mutations were originally identified), and taking the minimum value. We required that two cell types share at least three mutations meeting the same conditions as for computing normalized shared sSNV counts. We obtained 90% confidence intervals for the bootstrap estimate of the minMF.

#### Inferring snRNA-seq GluN/GABN clusters

We grouped single cells from our snRNA-seq and snATAC-seq datasets based on shared, identifiable somatic variants. We constructed a genotype matrix for 12381 total single cells (8279 in UMB4638 and 4102 in UMB4643) across both brains’ snRNA-seq and snATAC-seq datasets and applied Louvain clustering to identify 82 groups in UMB4638 and 83 in UMB4643, with cells in cluster sharing one or more common variants (Figure S8A). We estimated the cell type composition of each cluster to identify patterns using Seurat-derived annotations.

Given the coverage of somatic mutations in our single-cell transcriptomic and chromatin accessibility datasets, we sought to identify clusters of cells that share common sets of variants and identify their cell type compositions. We constructed an adjacency matrix that reports the number of variants shared by each pair of cells, and we applied Louvain clustering to identify groups of cells that share common variants. Each Louvain cluster represents a set of cells that shares a common set of variants and any other variants that might be represented exclusively within a subset of those cells.

Seurat’s annotations were used to mark the compositions of cell types within individual Louvain clusters. However, due to technical constraints on single-cell sequencing, significant variation exists in the number of cells within each cluster, between 2^1^-2^10^ and a significant number of clusters consisting of only a single cell. Thus, the estimated percentage of a cell type within a small cluster would be more prone to fluctuations in the cluster size than would an estimate for a large cluster. We employed empirical Bayes methods to generate an estimate of cell type compositions while controlling for cluster size and the number of variants represented in the cluster.

We focused on the proportions of GluNs and GABNs. We modeled the number of cells in each cluster of size N coming from a cell type as a beta-binomial random variable, in which the observed number of cells X depends upon parameters μ and σ. For each cell type, we used beta-binomial regression through the “aod” package to regress the X and N-X on the number of variants represented in the cluster and the log10 cluster size. This regression model yielded estimates of $\mu_{0}$ for each cluster and a shared $\sigma_{0}$, both of which were used to generate a prior distribution of cell type composition for each cluster. The posterior estimate of cell type composition was derived by computing $\frac{\alpha_{0}+X}{\alpha_{0}+\beta_{0}+N}$, where $\alpha_{0}=\frac{\mu_{0}}{\sigma_{0}}$ and $\beta_{0}=\frac{1-\mu_{0}}{\sigma_{0}}$.

#### Lineage and cell-type analysis with PRDD-seq

Lineage clading and cell-type analyses of UMB4638 and UMB4643 was completed using PRDD-seq, along with additional cell-type analysis using sSNVs in UMB4638 and UMB4643 and using designated marker genes used for cell-type and layer identification, all as previously described.^66^

### QUANTIFICATION AND STATISTICAL ANALYSIS

#### Definitions

Mosaic fractions (MFs) are defined as twice the alternate allele fraction (2 x AAF), expressed as an average if multiple amplicons in MIPP-seq were designed to target the sSNV. Following convention, we define mild and extreme outliers as observations that are respectively 1.5–3 and at least 3 interquartile ranges (IQRs) beyond the upper (q1) and lower (q3) quartile values. For reference, the IQR is measured as the difference between the lower and upper quartiles. Mathematically, mild outliers are observations (x) that satisfy x < q1 - [1.5,3]*IQR or x > q3 + [1.5,3]*IQR, whereas extreme outliers satisfy x < q1 - 3*IQR or x > q3 + 3*IQR, where IQR = q3-q1.

#### Statistical analysis

Statistical analysis, including counts, averages, and statistical tests are reported in figures and tables. Statistical analyses were performed in Microsoft Excel, Python, and R.

#### For Microsoft Excel and Python analyses

Analysis related to MF determination and background error rates are as previously described.^30^ Work was initially completed in Microsoft Excel and later adapted to an automated Python/Perl workflow that processed data in a high throughput manner. Briefly, all sites were first checked to ensure they meet the minimum QC metrics described above (e.g., >10,000X depth, and detected alternate allele matches expected alternate allele from WGS). Next, the AAF at the variant position was extracted for each amplicon, with the average and 95% confidence interval being calculated for each variant using the 2+ independent amplicons, if applicable. Next, background error rates for each amplicon were measured as the average AAF across the flanking 100 nts proximal to the target variant, and averages and confidence intervals for error rates were further calculated across replicate amplicons. Finally, the average AAFs of the targeted variant were directly compared against the average background errors using a t test. To ensure that error correction using Pollux did not introduce any errors in the data, assessments were performed using both the original (i.e., uncorrected) and error-corrected sequencing data.

#### Analyses of MIPP-seq data across cortex

Starting with an m-by-n MP matrix containing m mosaics and n tissues, we used the function “get_summary_stats” in the R-package “rstatix” to compute summary statistics for each mosaic, which include interquartile ranges (IQRs), and upper (q1) and lower (q3) quartile values. For reference, the IQR is measured as the difference between the q3 and q1. The outliers for each tissue were identified using the “identify_outliers” function in the R-package “rstatix”. Following convention, we define mild and extreme outliers as observations that are respectively 1.5–3 and at least 3 IQR beyond the upper (q1) and lower (q3) quartile values. Mathematically, mild outliers are observations (x) that satisfy x < q1 - [1.5,3]*IQR or x > q3 + [1.5,3]*IQR, whereas extreme outliers satisfy x < q1 - 3*IQR or x > q3 + 3*IQR, where IQR = q3-q1.

Because we consider the mosaic data to be paired across tissues, we considered using a 1-way repeated measures analysis of variance (ANOVA) using the functions “aov” (package “stats”). The four assumptions for ANOVA include 1) independence of observations, 2) no significant outliers, 3) normality, and 4) homogeneity of variances. Due to the tissue sampling methods and nature of SNVs, the observations were considered independent. Outliers were identified as above. Normality was verified via QQ plots (using function “ggqqplot” in R-packages “ggpubr” and “ggplot2”) and the Shapiro-Wilk test using the “shapiro_test” function in the R-package “rstatix”. Homogeneity of variances was verified (not shown in data) using the “levene_test” function in the R-package “rstatix”. As the above assumptions for ANOVA were violated, it was necessary to perform a non-parametric analysis of variance with Friedman’s test, by using the “friedman_test” function in the R-package “rstatix”.

Post hoc analysis was conducted for all tissue pairs, including biological replicates in BA9, BA18, and BA17. The “shapiro_test” function was once again used to determine if the difference in mosaic fractions between tissue pairs was normally distributed, to determine whether to use the “t.test” function (R-package “stats”) for the paired t test (if normally distributed) or the “wilcox.test” function (R-package “stats”) for the Wilcoxon Signed Rank test (if not normally distributed). The Benjamini-Hochberg procedure was performed to reduce the false discovery rate (FDR) in the multiple comparisons, by using the “p.adjust” function in the R-package “stats”. Q-values were computed using the “qvalue”.^116^

The correlation of mosaics between tissues was determined using the “cor” function in R-package “stats”. However, the paired nature of the mosaic fractions between tissues, and the wide variability of mosaic fraction values between sets of mosaics, resulted in spuriously high correlation coefficients. Future work will require normalization of data to account for the variability of mosaic fraction prior to performing correlation.

Additional R packages, UpsetR^117^ and core Tidyverse packages,^118^ were used for figure generation.

## Supplementary Material

1

2

3

4

5

6

7

Supplemental information can be found online at https://doi.org/10.1016/j.celrep.2025.116458.

## Figures and Tables

**Table:** KEY RESOURCES TABLE

REAGENT or RESOURCE	SOURCE	IDENTIFIER
Antibodies		
Mouse monoclonal anti-NeuN Alexa Fluor 488	Millipore	Clone A60; catalog number MAB377; RRID: AB_2149209
Biological samples		
Postmortem fresh-frozen human brain BA9 tissue	University of Maryland Brain & Tissue Bank; http://medschool.umaryland.edu/btbank/	UMB4638
Postmortem fresh-frozen human brain BA9 tissue	University of Maryland Brain & Tissue Bank; http://medschool.umaryland.edu/btbank/	UMB4643
Postmortem fresh-frozen human brain BA9 tissue	University of Maryland Brain & Tissue Bank; http://medschool.umaryland.edu/btbank/	UMB5575
Postmortem fresh-frozen human brain BA9 tissue	University of Maryland Brain & Tissue Bank; http://medschool.umaryland.edu/btbank/	UMB5580
Postmortem fresh-frozen human brain BA18 tissue	University of Maryland Brain & Tissue Bank; http://medschool.umaryland.edu/btbank/	UMB4638
Postmortem fresh-frozen human brain BA18 tissue	University of Maryland Brain & Tissue Bank; http://medschool.umaryland.edu/btbank/	UMB4643
Postmortem fresh-frozen human brain BA18 tissue	University of Maryland Brain & Tissue Bank; http://medschool.umaryland.edu/btbank/	UMB5575
Postmortem fresh-frozen human brain BA18 tissue	University of Maryland Brain & Tissue Bank; http://medschool.umaryland.edu/btbank/	UMB5580
Postmortem fresh-frozen human brain BA17 tissue	University of Maryland Brain & Tissue Bank; http://medschool.umaryland.edu/btbank/	UMB4638
Postmortem fresh-frozen human brain BA17 tissue	University of Maryland Brain & Tissue Bank; http://medschool.umaryland.edu/btbank/	UMB4643
Postmortem fresh-frozen human brain BA17 tissue	University of Maryland Brain & Tissue Bank; http://medschool.umaryland.edu/btbank/	UMB5575
Postmortem fresh-frozen human brain BA17 tissue	University of Maryland Brain & Tissue Bank; http://medschool.umaryland.edu/btbank/	UMB5580
Male fibroblast genomic DNA sample	Promega	G1471
Critical commercial assays		
QIAamp DNA mini kit	Qiagen	Cat. #51304
Illumina TruSeq PCR-free preparation kit	Illumina	20015962
AmpliSeq Primer Pool and Phusion U Mastermix	Thermo Fisher Scientific	F-562
Chromium Next GEM Single Cell 3' GEM, Library & Gel Bead Kits v3.1	10X Genomics	Catalog numbers PN-1000121 and PN-1000128
Deposited data		
210X WGS for UMB4638 and UMB4643 (BA17, BA18, and BA9)	Bizzotto et al.^31^	dbGaP: phs001485.v4.p1
210X WGS for UMB5575 and UMB5580 (BA17, BA18, and BA9)	This study	dbGaP: phs001485.v4.p1
MIPP-seq data for UMB4638 and UMB4643	This study	dbGaP: phs001485.v4.p1
Panel single-cell MDA for UMB4638 and UMB4643	This study	dbGaP: phs001485.v4.p1
Deposited single-nucleus RNA-seq for UMB4638 and UMB4643 BA17/BA18	This study	dbGaP phs001485.v4.p1
Deposited single-nucleus RNA-seq for UMB4638 and UMB4643 BA9	Bizzotto et al.^31^	dbGaP: phs001485.v4.p1
Oligonucleotides		
Primers	This paper (see “STAR Methods”)	N/A
Software and algorithms		
Bwa-mem	Li.^87^	v0.7.8
Genome Analysis Toolkit (GATK)	McKenna et al.^88^	v3.5 (WGS) and v3.7 (MIPP-seq)
Mutect2	Benjamin et al^89^	version nightly-2016-04-25-g7a7b7cd
MosaicForecast	Dou et al.^37^	https://github.com/parklab/MosaicForecast
CutAdapt	Martin^90^	https://cutadapt.readthedocs.io/en/stable/
samtools	Li^91^	v1.3.1
bedtools	Quinlan and Hall^92^	v2.26.0
FASTX	v0.0.14	http://hannonlab.cshl.edu/fastx_toolkit/
Pollux	Marinier et al.^93^	1.0.2
bamclipper	Au et al.^94^	https://anaconda.org/bioconda/bamclipper
scistree	Wu^95^	https://github.com/yufengwudcs/ScisTree
mpboot	Hoang et al.^96^	https://github.com/diepthihoang/mpboot
CellRanger	10X Genomics	v7.0.1
DropletQC	Muskovic et al.^97^	https://github.com/powellgenomicslab/DropletQC
Seurat	Hao et al.^98^	v5.0.1
CellBender	Fleming et al.^99^	v0.3.0
Analysis software for WGS, snRNA-seq, and pscMDA	This study	https://github.com/parklab/spatial_sampling_analysis and https://doi.org/10.5281/zenodo.17138985
Analysis software for MIPP-seq	This study	https://doi.org/10.5281/zenodo.17109324 and https://github.com/soniankim/brain-clone-mosaic
Other		
GRCh37 reference genome	1000 Genomes	https://www.internationalgenome.org/data/
gnomAD	Broad Institute	https://gnomad.broadinstitute.org/

## Highlights

- Somatic mutations track human cortical lineages across the BA17/18 border
- Clones across the BA17/18 border show asymmetric allocation and developmental fates
- Restricted dispersion across BA17/18 differs from broad frontoparietal dispersion
- Mutations in snRNA-seq highlight late-diverging GluN and GABAergic neuronal clones
