# Supplementary material for: Combined somatic mutation and transcriptome analysis reveals region-specific differences in clonal architecture in human cortex
Source: Cell Rep. Author manuscript; Available in PMC 2026 Jan 5. (PMC12766648; doi:10.1016/j.celrep.2025.116458)
Supplement: 1 [file NIHMS2125806-supplement-1.pdf]

Cell Reports, Volume *44*

Supplemental information

Combined somatic mutation and transcriptome analysis reveals region-specific differences in clonal architecture in human cortex

Vinayak V. Viswanadham, Sonia N. Kim, Emre Caglayan, Ryan N. Doan, Yanmei Dou, Sara Bizzotto, Sattar Khoshkhoo, August Yue Huang, Rebecca Yeh, Brian H. Chhouk, Alex Truong, Kathleen M. Chappell, Marc Beaudin, Alison Barton, Shyam K. Akula, Yifan Zhao, Lariza Rento, Michael Lodato, Ryan A. Szeto, Javier Ganz, Pengpeng Li, Jessica W. Tsai, Robert Sean Hill, Peter J. Park, and Christopher A. Walsh

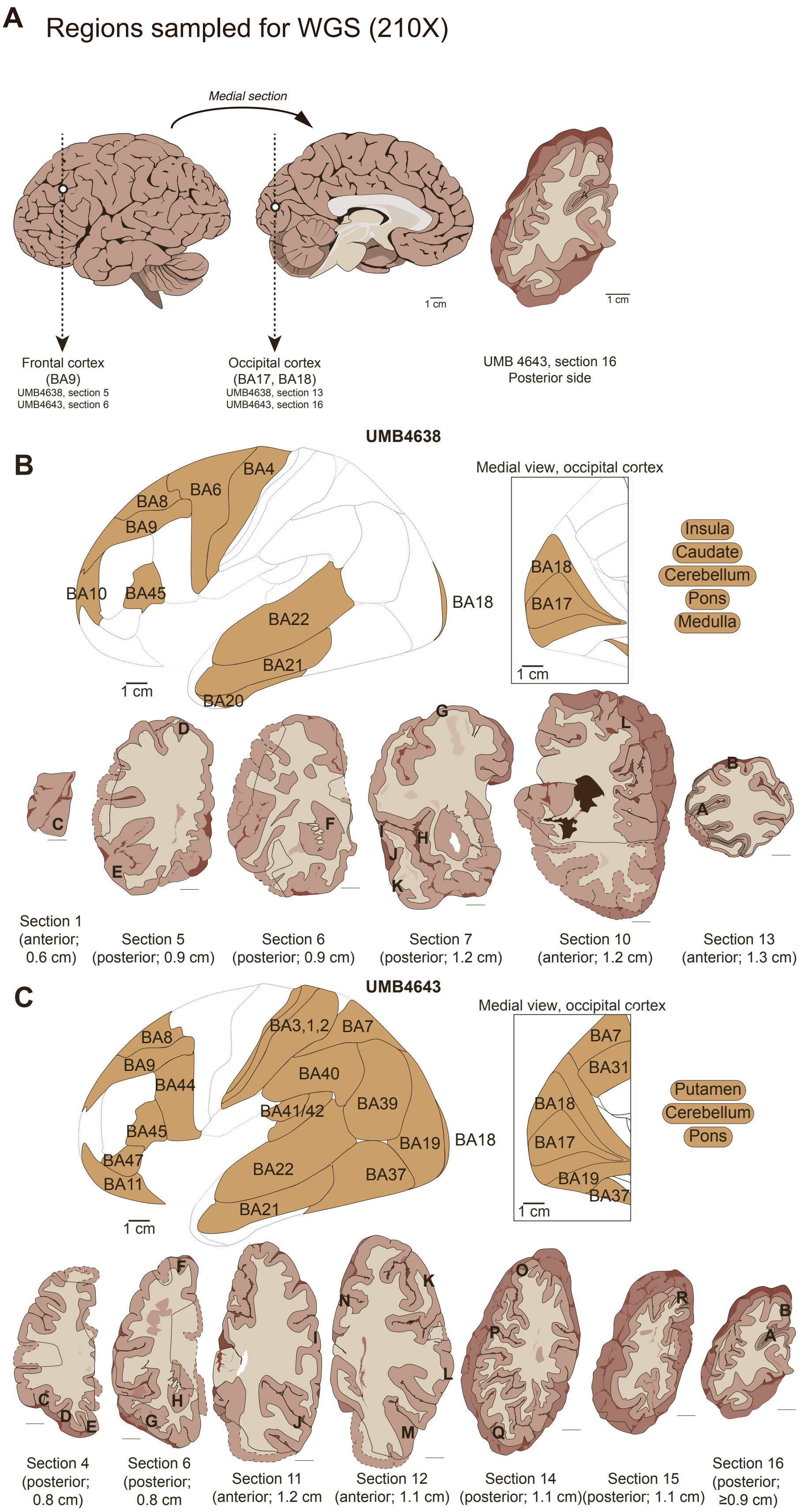
A
Regions sampled for WGS (210X)
Medial section
1 cm
1 cm
Frontal cortex
(BA9)
UMB4638, section 5
UMB4643, section 6
Occipital cortex
(BA17, BA18)
UMB4638, section 13
UMB4643, section 16
UMB 4643, section 16
Posterior side
UMB4638
B
BA4
BA6
BA8
BA9
BA10
BA45
BA22
BA21
BA20
BA18
1 cm
Medial view, occipital cortex
BA18
BA17
1 cm
Insula
Caudate
Cerebellum
Pons
Medulla
Section 1
(anterior;
0.6 cm)
Section 5
(posterior; 0.9 cm)
Section 6
(posterior; 0.9 cm)
Section 7
(posterior; 1.2 cm)
Section 10
(anterior; 1.2 cm)
Section 13
(anterior; 1.3 cm)
UMB4643
C
BA8
BA9
BA44
BA45
BA47
BA11
BA3,1,2
BA7
BA40
BA41/42
BA39
BA19
BA22
BA37
BA21
BA18
1 cm
Medial view, occipital cortex
BA7
BA31
BA18
BA17
BA19
BA37
1 cm
Putamen
Cerebellum
Pons
Section 4
(posterior;
0.8 cm)
Section 6
(posterior;
0.8 cm
Section 11
(anterior; 1.2 cm
Section 12
(anterior; 1.1 cm)
Section 14
(posterior; 1.1 cm)
Section 15
(posterior; 1.1 cm)
Section 16
(posterior;
≥0.9 cm)

Supplementary Figure 1: Locations of cortical, subcortical, and non-cortical brain samples used for mosaic characterization and distribution analysis in MIPP-seq and WGS. Related to Figures 1 and 2.
(A) For 210X WGS, DNA was prepared and sequenced from the frontal (BA9) and occipital cortex (BA18 and BA17). Tracings of the occipital cortex section show sampling of adjacent cortical areas: primary visual cortex, BA17 ("A") indicated by the stria of Gennari (white line); and secondary visual cortex, BA18 ("B"). Locations of samples from UMB4638 **(B)** and UMB4643 **(C)**. For each panel, the top half shows the cortical brain map of the left hemisphere with BA annotations (see methods) indicate which BAs are represented in the study. Shaded regions indicate the BA represented by the obtained tissue biopsy. Some regions were unavailable due to prior sampling of these brains; these are indicated by the non-shaded regions with dashed grey lines. The bottom half shows lucida tracings of sampled cortical sections (see methods) indicate the BA sampled from that coronal section. Viewing orientation and the thickness of the coronal section are listed below each section. All sections are scaled; scale bar is 1 cm. **(B)** Sampled tissues (in UMB4638) are listed as: (C) BA10; (D) BA8; (E) BA45; (F) Caudate nucleus; (G) BA6; (H) Insula; (I) BA22; (J) BA21; (K) BA20; (L) BA4; (B) BA18; and (A) BA17. BA9 (not marked) is from section 5. Not shown here are additional brain tissues: pons, medulla, and cerebellum. **(C)** Sampled tissues (in UMB4643) are listed as: (C) BA45; (D) BA47; (E) BA11; (F) BA8; (G) BA44; (H) Putamen; (I) BA40; (J) BA41/42; (K) BA3/1/2; (L) BA22; (M) BA21; (N) BA31; (O) BA7; (P) BA39; (Q) BA37; (R) BA19; (B) BA18; and (A) BA17. BA9 (not marked) is from section 6. Not shown here are additional brain tissues: pons and cerebellum.

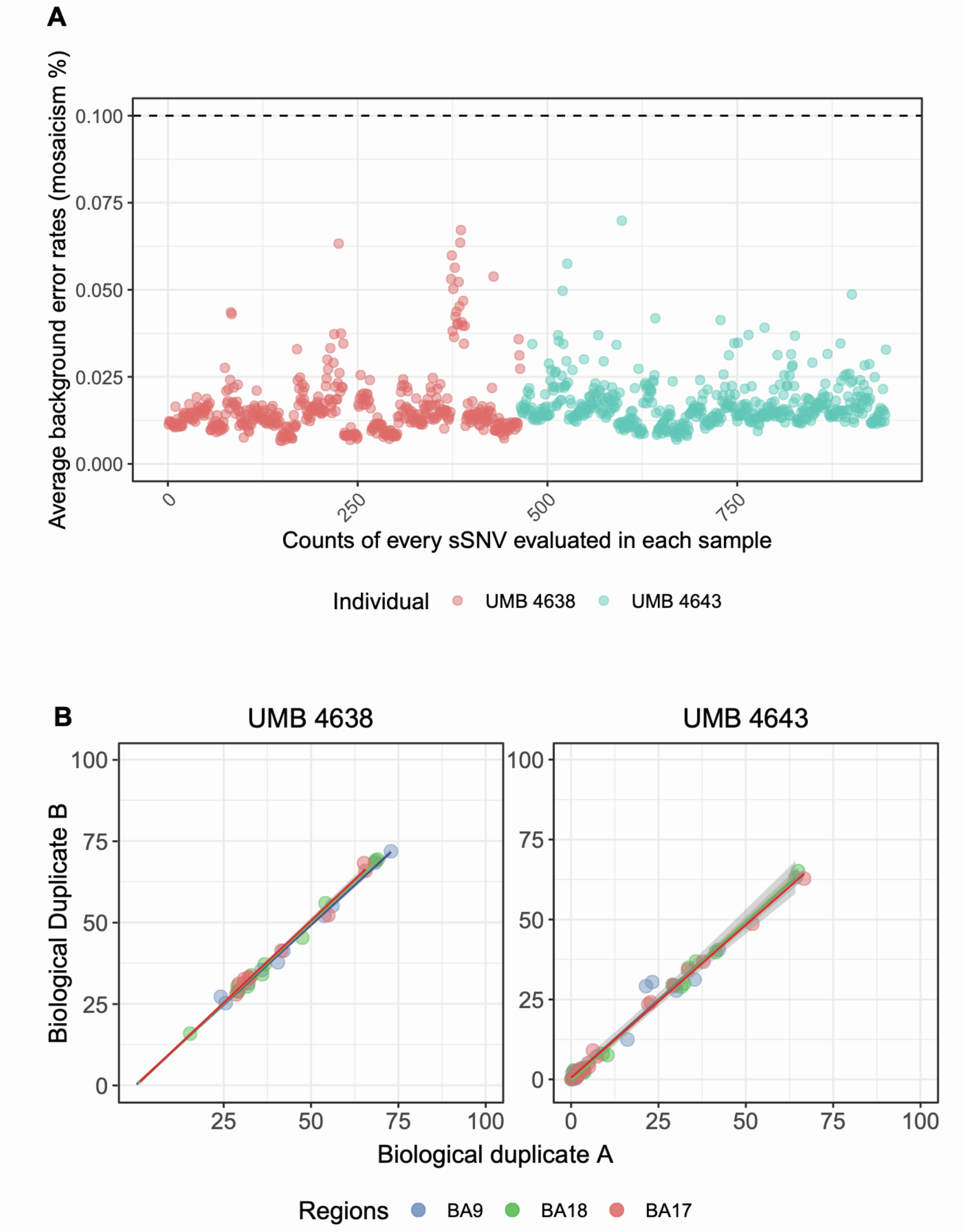

Supplementary Figure 2: Benchmarking the mosaic fraction estimates of MIPP-seq. Related to Figure 3.
(A) Average background error rate for sSNVs in each cortical region using MIPP-seq for UMB4638 and UMB4643. **(B)** Correlation of MIPP-seq mosaic fraction estimate across two biological duplicates (A and B) taken for each cortical region.

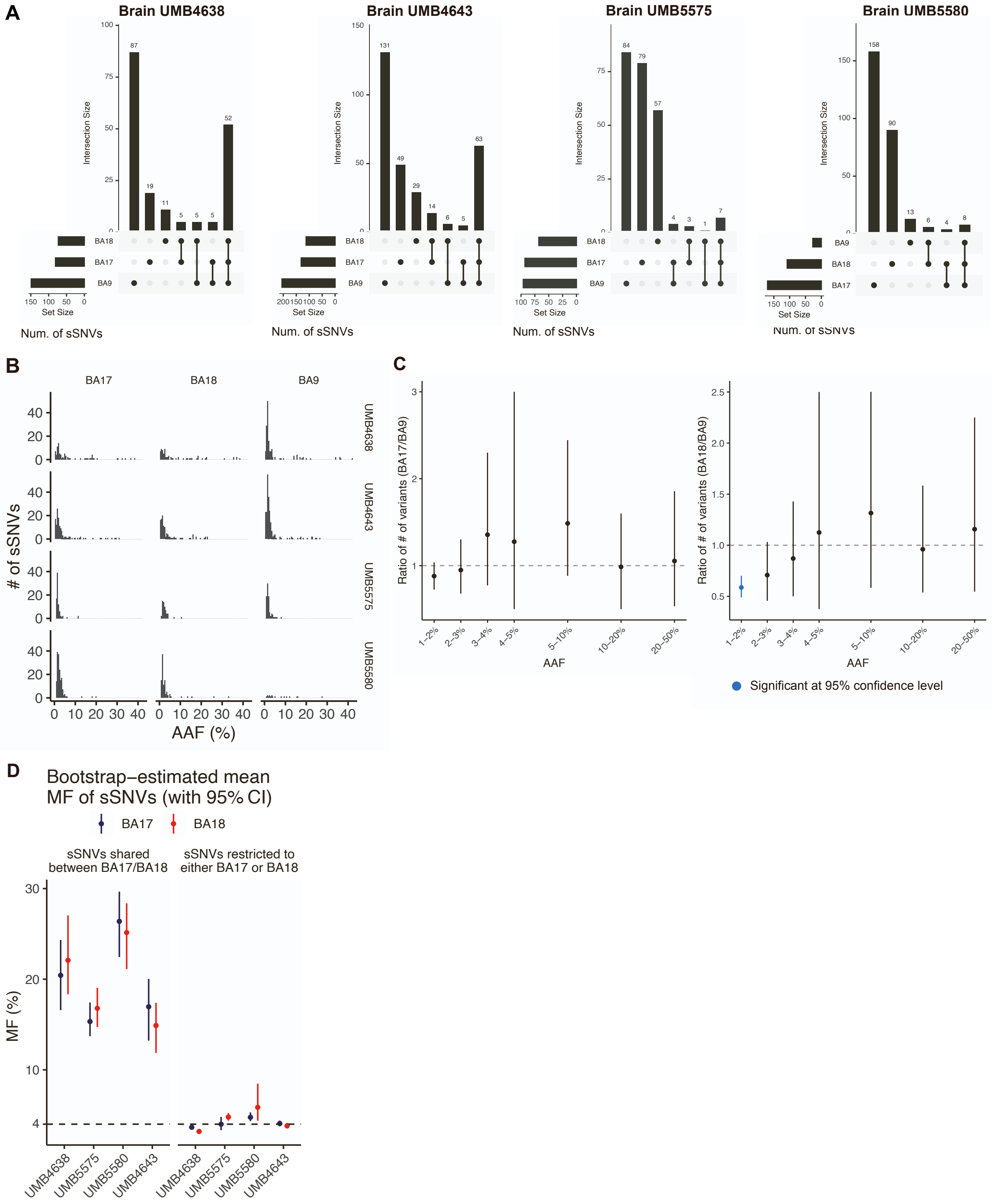

Supplementary Figure 3: Differences in sSNV counts across different cortical regions. Related to Figure 2. **(A)** Overall number of mutations called in >200X WGS from UMB 4638, UMB4643. UMB5575 and UMB 5580, and categorization of the distribution of each called variant. **(B)** Allele frequencies of sSNVs identified per brain and per cortical region. **(C)** Ratio of the sSNV count between BA17 and BA9 (”BA17/BA9”), and BA18 and BA9 (”BA18/BA9”) for each alternate allele frequency range. (**D)** Bootstrap estimates of the average AAFs of regionally restricted or shared variants (between BA17 and BA18). Differences between shared-region and restricted-region variants’ AAFs are statistically significant (p<1e-2, two-sided t-test) for each donor.

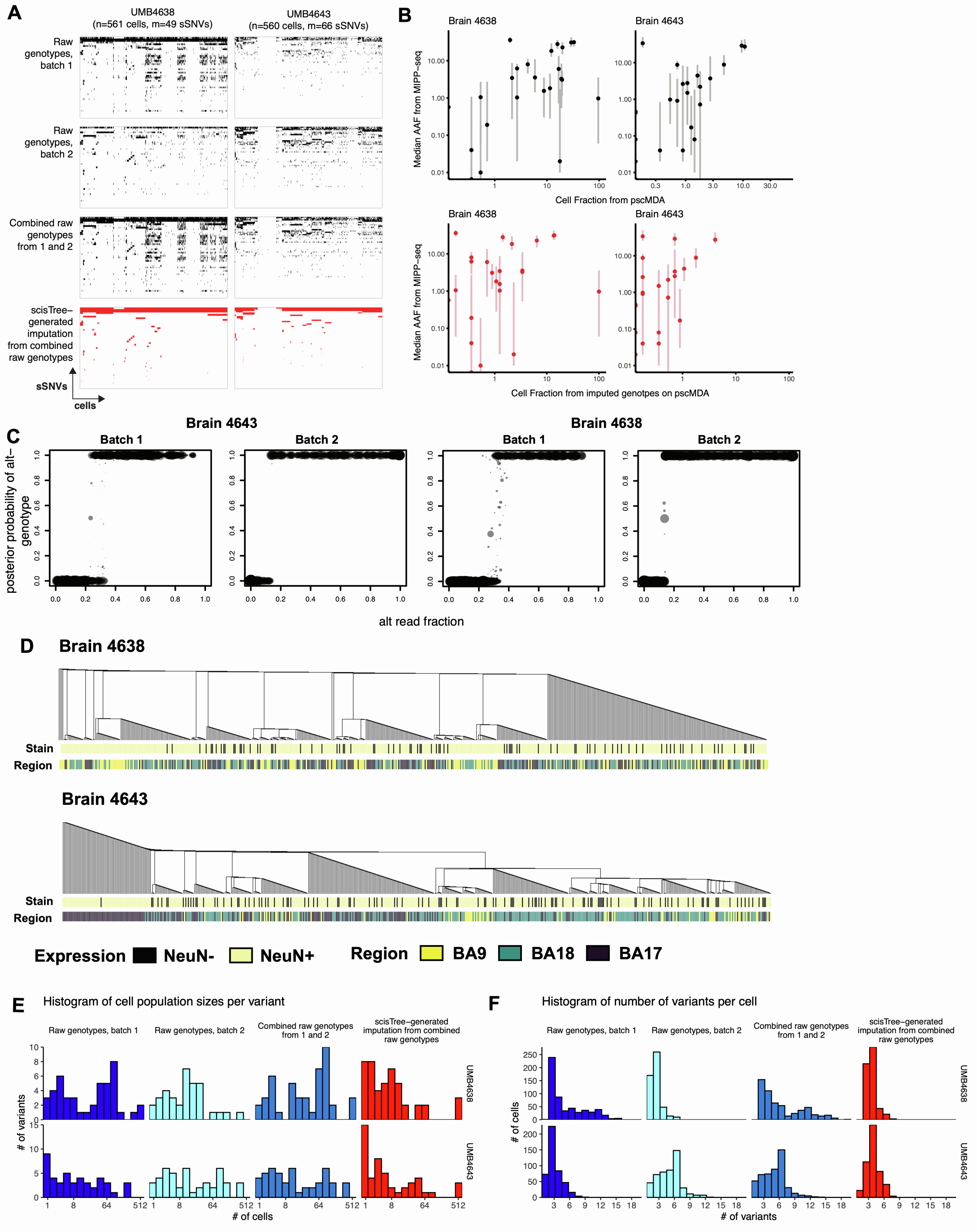

A
UMB4638
(n=561 cells, m=49 sSNVs)
UMB4643
(n=560 cells, m=66 sSNVs)
Raw genotypes, batch 1
Raw genotypes, batch 2
Combined raw genotypes from 1 and 2
scisTree–generated imputation from combined raw genotypes
sSNVs
cells
B
Brain 4638
Brain 4643
Median AAF from MIPP–seq
Cell Fraction from pscMDA
Cell Fraction from imputed genotpes on pscMDA
C
Brain 4643
Brain 4638
Batch 1
Batch 2
posterior probability of alt–genotype
alt read fraction
D
Brain 4638
Brain 4643
Stain
Region
Expression
NeuN-
NeuN+
Region
BA9
BA18
BA17
E
Histogram of cell population sizes per variant
Raw genotypes, batch 1
Raw genotypes, batch 2
Combined raw genotypes from 1 and 2
scisTree–generated imputation from combined raw genotypes
UMB4638
UMB4643
# of variants
# of cells
F
Histogram of number of variants per cell
# of cells
# of variants

Supplementary Figure 4: Construction of single-cell lineage trees and inference of lineage parameters for UMB4638 and UMB4643. Related to Figure 4.

(A) Genotype matrix of 561 (UMB4638, left) and 560 (UMB4643, right) single cells. Cells were sequenced using panel single-cell multiple displacement amplification (pscMDA) at select somatic mutation sites previously identified from 210X WGS and validated using MIPP-seq. Cells were sequenced across two batches, with each individual batch (top two heatmaps) genotyped before integration of genotypes (third heatmap from top). Shown are the integrated genotype matrices. scistree [105] was used to impute genotypes for single cells (bottom-most heatmap) to ensure that all single cells' genotypes are compatible with the infinite sites assumption while ensuring that high-confidence genotypes are retained and that the phylogeny of the single cells is retained as much as possible. In each matrix, hierarchical clustering of the cells and variants was conducted independently of other matrices. **(B)** Comparisons of mosaic (cell) fractions inferred by pscMDA and MIPP-seq. Mosaic fractions inferred from the integrated genotype matrix (top, black) and the post-imputation genotype matrix (bottom, red) of pscMDA are plotted against the alternate allele fractions (AAFs) from MIPP-seq (IonTorrent- based sequencing of candidate somatic variants). For some variants, MIPP-seq variants are too low to confidently distinguish above error read fractions. Vertical bars (top, grey; bottom, pink) indicate the range of MIPP-seq AAFs across 21 brain regions and structures where the variant was sequenced in the corresponding individual, with points' y-values reporting the median across tissues. **(C)** Posterior probability of cells carrying the somatic-alt allele at each site. Each point represents a site within a specific cell. Points are sized by the log10 total coverage (with the log-value divided by 100 to determine point size). **(D)** Phylogenetic trees (represented as cladograms) constructed from pscMDA cells. The heatmaps below the tree mark the nucleus stain (NeuN+/-) and region (BA17, BA18, BA9) from which the variant originates. **(E)** Distributions of the numbers of cells carrying each sSNV shown for each batch, the combination of the 2 batches, and the imputed dataset. **(F)** Distributions of the numbers of sSNVs within each cell are shown for each batch, the combination of the 2 batches, and the imputed dataset.

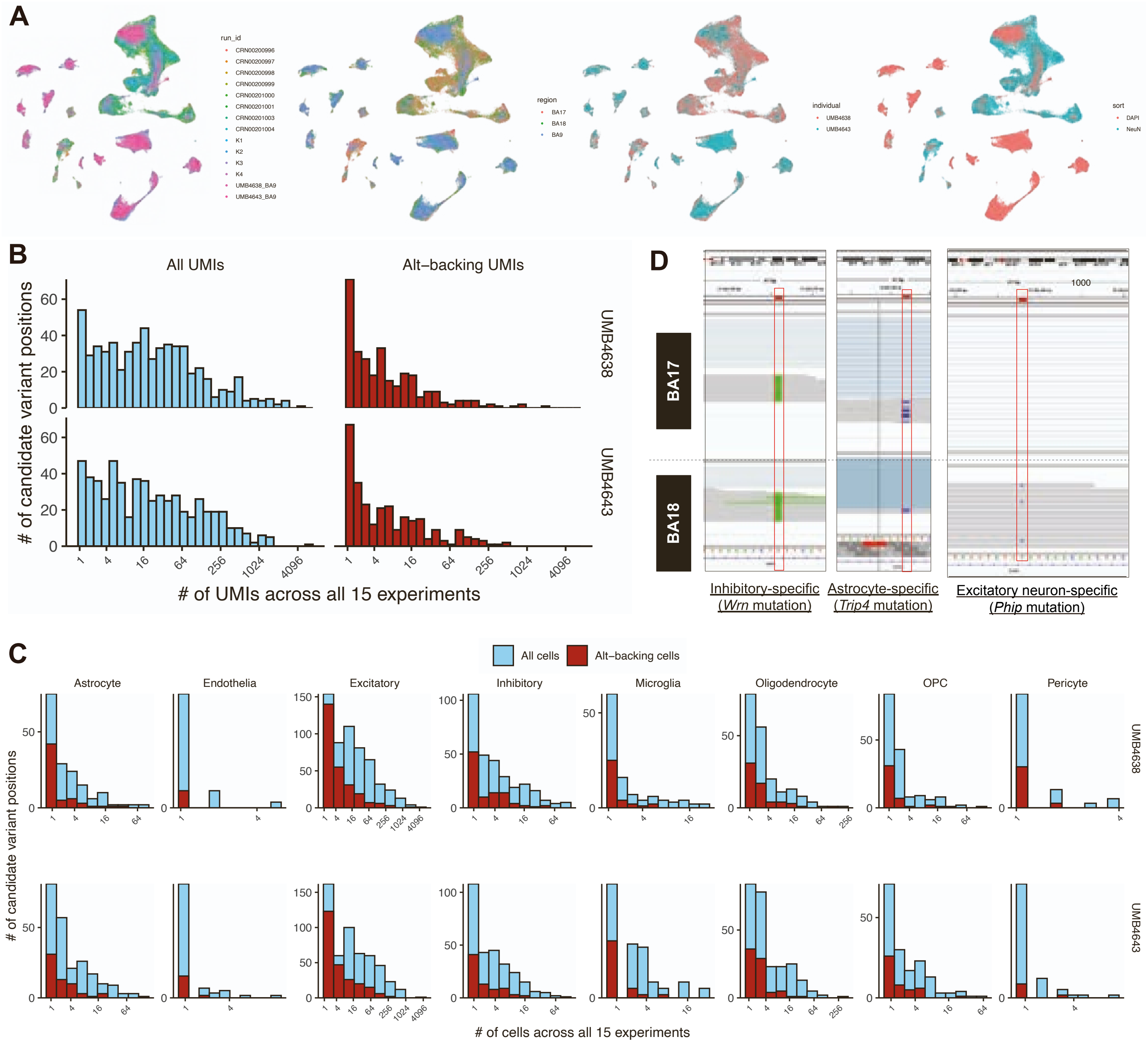

Supplementary Figure 5: Coverage statistics and basic properties of sSNVs captured in snRNA-seq. Related to Figures 5 and 6.

(A) UMAPs of 71,461 nuclei from snRNA-seq colored by individual, sorting strategies, regions, and experiment. **(B)** Log-scaled number of all UMIs and alt UMIs per test sSNV over all 15 experiments studied in snRNA-seq. **(C)** Log-scaled number of all and alt UMIs per test sSNV over all 15 experiments studied in snRNA-seq divided by broad cell type. **(D)** IgV plots of example variants captured in inhibitory neurons, astrocytes, and excitatory neurons.

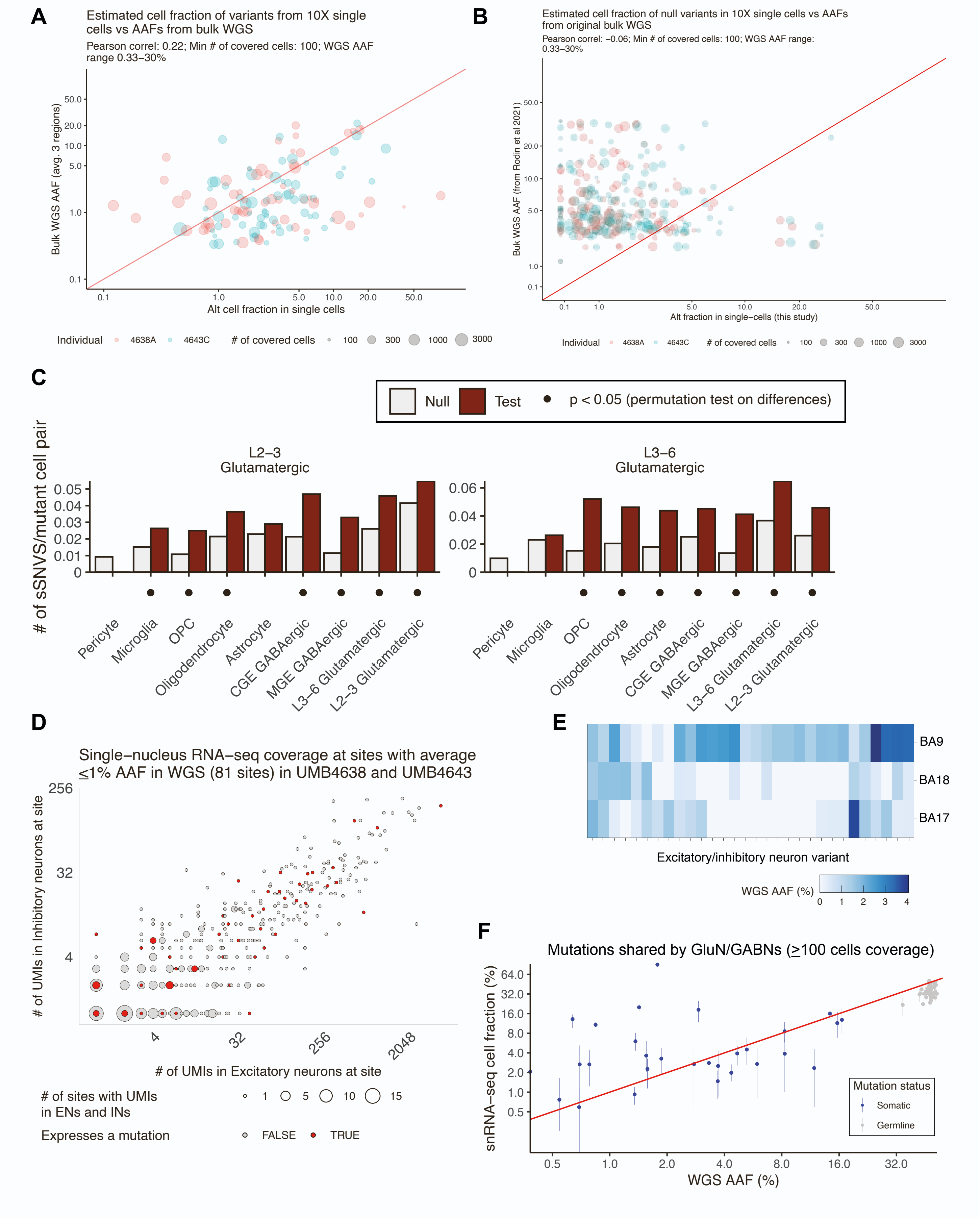

A
Estimated cell fraction of variants from 10X single cells vs AAFs from bulk WGS
Pearson correl: 0.22; Min # of covered cells: 100; WGS AAF range 0.33–30%
Bulk WGS AAF (avg. 3 regions)
Alt cell fraction in single cells
Individual 4638A 4643C
# of covered cells 100 300 1000 3000
B
Estimated cell fraction of null variants in 10X single cells vs AAFs from original bulk WGS
Pearson correl: −0.06; Min # of covered cells: 100; WGS AAF range: 0.33–30%
Bulk WGS AAF (from Rodin et al 2021)
Alt fraction in single−cells (this study)
Individual 4638A 4643C
# of covered cells 100 300 1000 3000
C
Null
Test
p < 0.05 (permutation test on differences)
# of sSNVS/mutant cell pair
L2–3 Glutamatergic
L3–6 Glutamatergic
Pericyte
Microglia
OPC
Oligodendrocyte
Astrocyte
CGE GABAergic
MGE GABAergic
L3–6 Glutamatergic
L2–3 Glutamatergic
D
Single−nucleus RNA−seq coverage at sites with average ≤1% AAF in WGS (81 sites) in UMB4638 and UMB4643
# of UMIs in Inhibitory neurons at site
# of UMIs in Excitatory neurons at site
# of sites with UMIs in ENs and INs 1 5 10 15
Expresses a mutation FALSE TRUE
E
BA9
BA18
BA17
Excitatory/inhibitory neuron variant
WGS AAF (%)
F
Mutations shared by GluN/GABNs (≥100 cells coverage)
snRNA−seq cell fraction (%)
WGS AAF (%)
Mutation status
Somatic
Germline

Supplementary Figure 6: Extent of clonal sSNV sharing between glutamatergic and GABAergic neurons. Related to Figure 5.

(A and B) Correlation of the mosaic (cell) fraction of each sSNV (at <50% AAF) in snRNA-seq with the mean AAF from WGS for test (sample-matched) **(A)** and null (from different samples than were studied) (**B)** sSNVs. For each sSNV's mosaic fraction calculation, only cells that have coverage at the locus are considered in the denominator. (**C)** Normalized number of sSNVs shared between glutamatergic neurons (GluNs) and different cell types. To normalize the number of sSNVs, the number of sSNVs found shared between cells from two different cell types is divided by the number of pairs of cells sharing the mutation in which each pair consists of cells from different type. Normalization is conducted to adjust for the number of cell pairs comprising cell types of different abundances in our dataset. Normalized sSNV counts are obtained for "test" mutations (dark red: sSNVs detected in UMB4638 and UMB4643 WGS and corroborated in sample-matched snRNA-seq) and for "null" mutations (grey: sSNVs detected from unrelated individuals [38] whose alternate alleles were detected in UMB4638 and UMB4643 snRNA-seq). Statistical significance was computed for the differences in normalized sSNV counts between test and null mutations using a permutation test (**Methods**). **(D)** The number of UMIs (i.e., transcript counts) detected across single GluNs and GABAergic neurons (GABNs) from snRNA-seq at sites with mutant alleles detected at <1% AAF in WGS data. If a site's UMIs support the presence of mutant alleles, points are colored red. Points are sized by the number of sites with the observed pairing of GluN and GABN UMI counts. **(E)** A heatmap of the WGS AAFs of 32 sSNVs at average MFs of ≤8% (≤4% AAF) that were found detected in both GluNs and GABNs. **(F)** Correlation of snRNA-seq-based mosaic fraction and WGS AAF for sSNVs tagging mixed GluN/GABN clones. The sSNVs shown have ≥100 cells coverage.

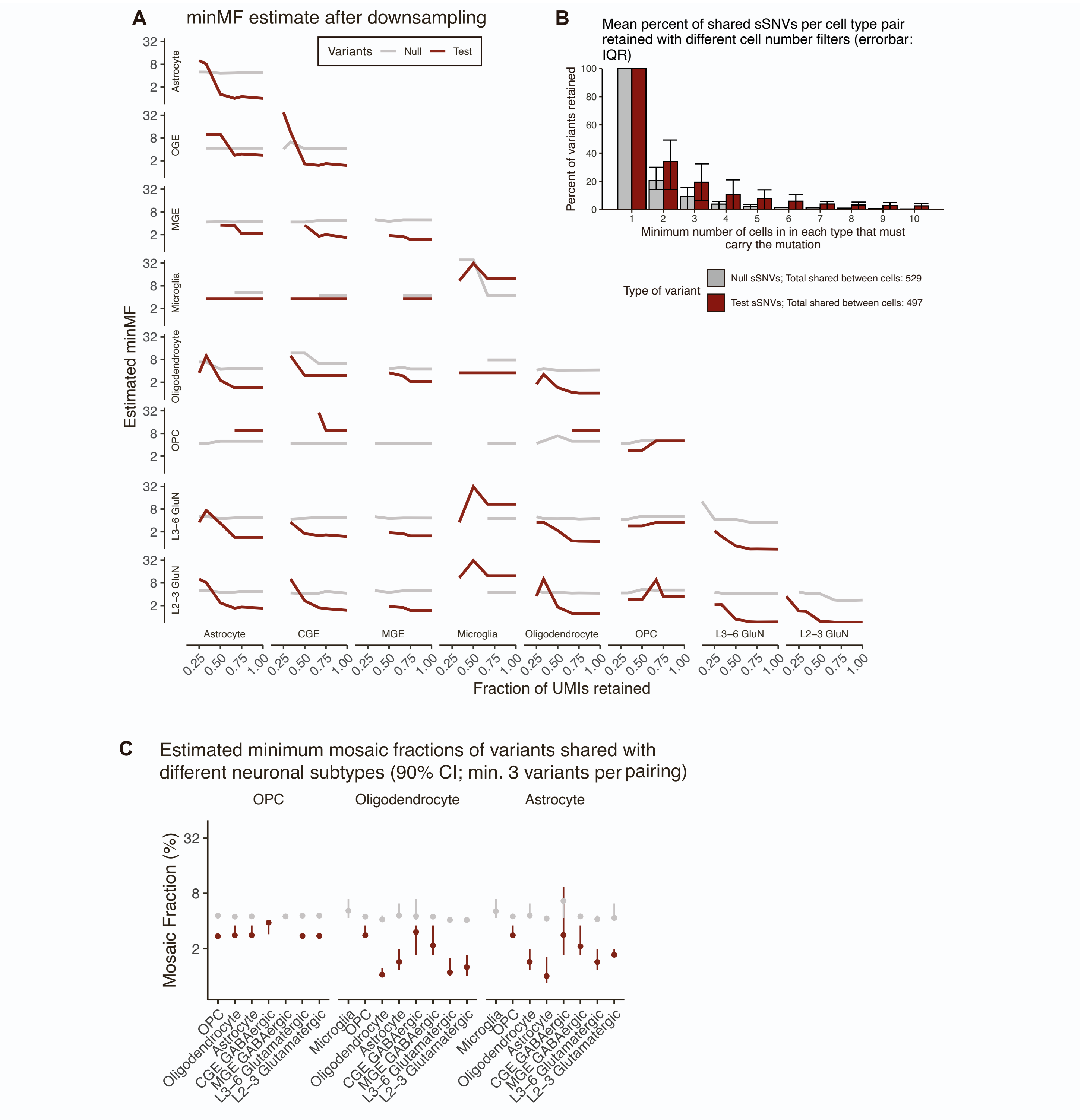

Supplementary Figure 7: Properties of minimum mosaic fraction (minMF) estimates. Related to Figure 5.

(A) The robustness of the minMF statistic to downsampling UMIs from UMB4638 and UMB4643 snRNA-seq data. Each plot shown in the matrix represents the minMF statistic computed for two sets of variants, one sample-matched to the snRNA-seq data (blue) and one from unmatched individuals (as described in **Figure S7** and **Methods**). The minMF for sample-matched variants is estimated to be stable in datasets comprising approximately 50% of the observed number of UMIs. **(B)** The percent of shared sSNVs per cell type pair retained with different filters for the number of cells per cell type that must report the alt-allele of the sSNV. **(C)** The minMF of variants shared with macroglia and different cell types. The low minMFs amongst oligodendrocytes and amongst astrocytes are suggestive of mutations being confined to cell type-specific lineages.

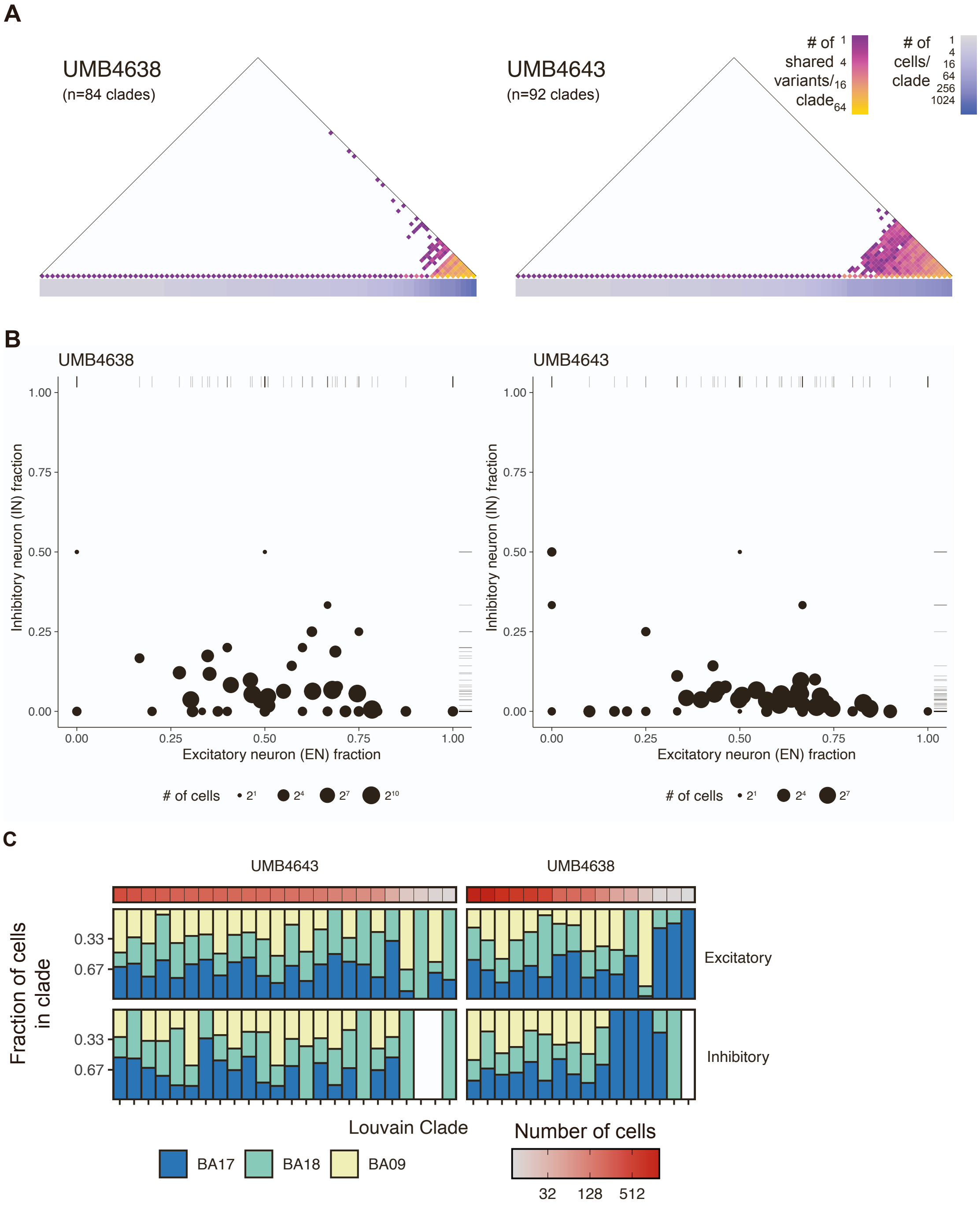

Supplementary Figure 8: Subclonal structure based on shared sSNVs in snRNA-seq data. Related to Figure 7.

(A) Groups of cells that are found to share mutations in snRNA-seq data. Groups ("clades") are arranged along the diagonal of the half-square, with off-diagonal entries colored by the number of variants mutually shared between groups. **(B)** Observed fractions of cells in variant-sharing subgroups that are annotated as excitatory or inhibitory neurons. These raw fractions, along with cell numbers and experimental metadata, were used to generate Empirical Bayes estimates of the cellular composition of each subgroup after controlling for biological and technical factors **(Figure S5A)**.

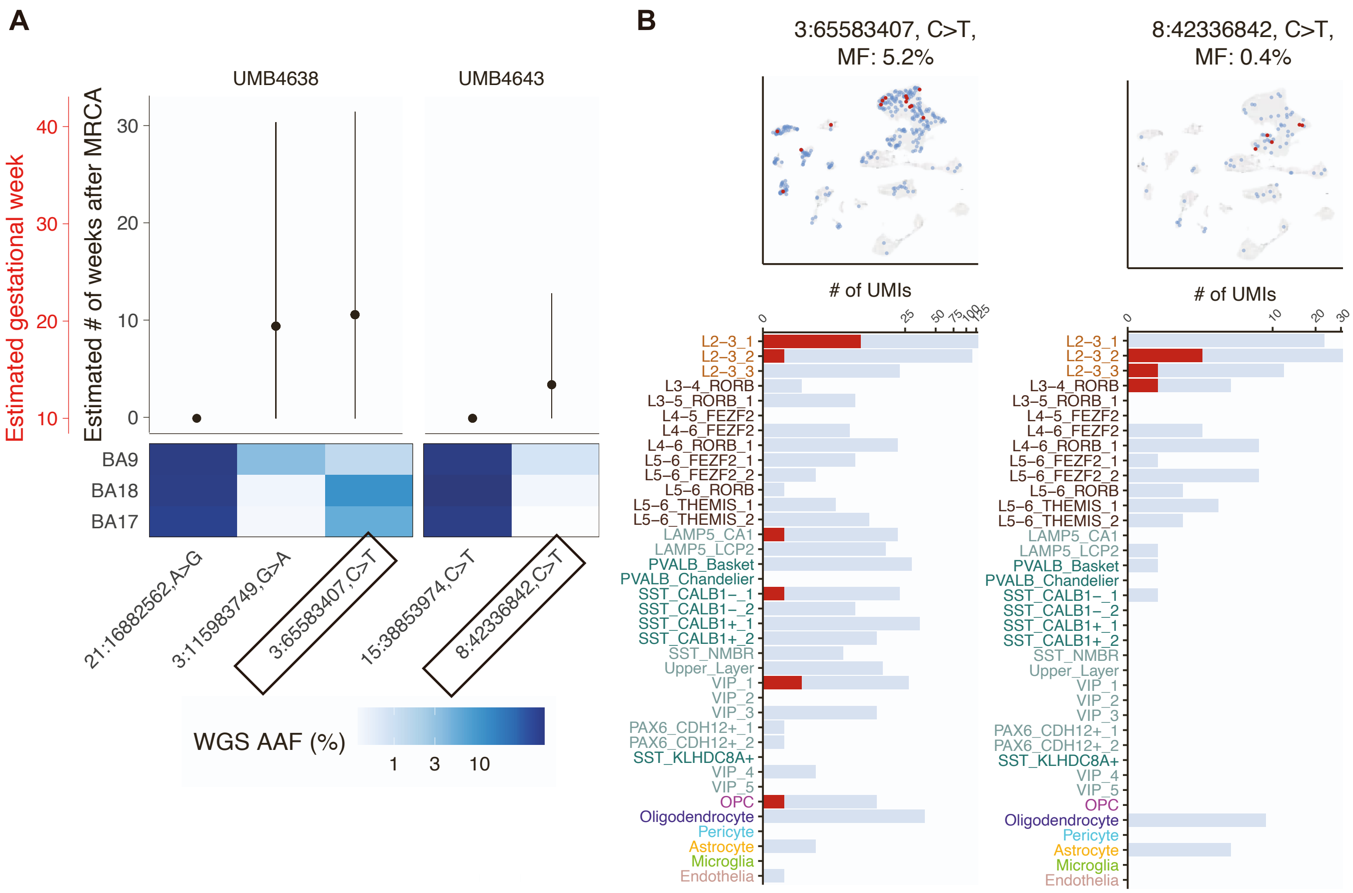

Supplementary Figure 9: Estimated timing of snRNA-seq detected sSNVs based on overlaps with single-cell lineage trees. Related to Figure 7.

Estimated time-of-origins and spatial distributions of variants marking different cell types. **(A)** Time-of-origin estimates given in PMW (black) or GW (red) for select variants identified in different cell types in UMB4638 (left) and UMB4643 (right). **(B)** Heatmaps showing the WGS AAF estimates for variants in different regions are also shown. UMAPs and the cell type histogram for 8:42336842, C>T, an upper/middle-layer-restricted GluN, is shown.
