# Supplementary material for: Combined somatic mutation and transcriptome analysis reveals region-specific differences in clonal architecture in human cortex
Source: Cell Rep. Author manuscript; Available in PMC 2026 Jan 5. (PMC12766648; doi:10.1016/j.celrep.2025.116458)
Supplement: 2 [file NIHMS2125806-supplement-2.pdf]

Supplemental Methods

Fitting the coalescent model

Assumptions underlying the model

The two individuals contributing UMB4638 and UMB4643 were neurotypical and had no apparent significant cortical malformations, and the somatic mutations we identified from these individuals are neutral (i.e. no evidence for positive or negative selection at the loci where alleles were discovered). We make the following assumptions:

1. The variants arise by neutral evolution, and the population follows a Wright-Fisher model (Tran et al., 2013).
2. The effective population size (i.e., the expected overall number of cells contributing to the mutations in the lineage) is constant across generations during the development of the lineage but must be inferred during model fitting.
3. The mutation rate is similar across our three studied regions.
4. Divisions are symmetric; each cell yields two identical progeny.
5. Cell divisions are independent of and do not influence one another.

For the first and second assumptions, the Wright-Fisher model is a convenient framework to model how newly-arisen mutations are passed down to successive generations by genetic drift and in the absence of selection, recombination, or gene flow. Furthermore, although the cell population expands during the development of the cortex, the effective population size is likely much smaller than the entire population of the developing cortex, and the total population size may fluctuate in significant ways that we cannot model with our existing data. A Wright-Fisher model treats the effective population size as constant but as a parameter to be inferred, which can simplify computations. For the third assumption, regional differences in the mutation rates would create significant differences in average mutation frequencies (Gillespie, 1998). However, we did not observe statistically significant differences in the average allele frequencies of rare mosaic variants across the three regions (**Fig. S3**). The fourth and fifth assumptions can be made because we are studying the early clonal development of the cortex and not collecting specific information about the cell type identities of the cells in our lineage tree. Although we can distinguish cells by NeuN staining, we do not have any more explicit information to model cell type hierarchies or interactions.

Description of the model and the method used to fit parameters

We adapted the standard Kingman coalescent model (Kingman, 1982) to conduct inference. Equations to estimate the coalescence times of the entire population and the individual depend upon a number of parameters:

1. The population size N.
2. The number of segregating sites S, a subset of the total observed loci ℓ which varies across the cells and thus gives lineage information.
3. The number of sampled cells n.
4. The mutation rate μ (per base per cell per division).
5. A phylogenetic tree ψ constructed using the observed mutations.

A coalescent model is used to describe how mutations within a population originated from a common ancestor over time. We model how long it would take for cells of sample size n to "coalesce" to their single common ancestor as we look backward in time. Specifically, the "coalescence time" is the time elapsed between two consecutive coalescence events j and $j-1$ when the population shrinks from n_j to n_{j-1} as two of the n_j cells coalesce to their most recent common ancestor (MRCA), decreasing the population size by 1. For a large population, the coalescence time T_j of two cells can be modeled as an exponentially distributed variable

$$T_j \sim \text{Exponential}\left(\frac{n_j(n_j-1)}{2}\right) \tag{S1}$$

$$\mathbb{E}[T_j] = \frac{2}{n_j(n_j-1)} \tag{S2}$$

where $\mathbb{E}[T_j]$, the expected value of the coalescence time, is the probability that the two cells randomly selected from the population share a common ancestor.

The estimated coalescence time of the entire population is

$$T = \sum_{j=1}^{n} T_j \tag{S3}$$

$$\mathbb{E}[T] = \sum_{j=1}^{n} \mathbb{E}[T_J] = \sum_{j=1}^{n} \frac{2}{n_j(n_j - 1)} \tag{S4}$$

The expected value of the coalescence time, which is obtained by the linearity of expectation, is a summary statistic of the average time for the sampled set of cells to coalesce into their MRCA. The phylogenetic tree ψ can be built from such a sampled population of n cells with S observed segregating sites, i.e., mutations whose genotypes differ amongst the cells and could therefore be mapped to the branches of the tree. If the mutation rate per cell is μ, then $\theta = 2N\mu\ell$ represents the population-wide mutation rate (i.e. the number of new mutations entering the population in a given generation) over our ℓ positions. Given that mutations accumulate along branches of ψ (representing the accumulation of mutations in the lineage), the sum of branch lengths in ψ is

$$L = \sum_{j=1}^{n} n_j T_j \tag{S5}$$

where population is of size n_j at time j (at $j = n$, $n_n = n$). The expected sum of branch lengths, $\mathbb{E}[L]$, represents another summary statistic that we can infer about our sample.

Acceptable values of our parameters $\Omega = \{\mathbb{E}[T], \mathbb{E}[L], N, \mu\}$ will yield a high likelihood for the observed value of S, and we seek to generate posterior distributions for our parameters to describe the timing of the lineage process. We used rejection sampling (Tavaré et al., 1997; Beaumont et al., 2002; Csilléry et al., 2010) to determine values of our parameters Ω by computing the following in sequence:

$$\mu = 10^{-9} \tag{S6}$$

$$\eta \sim \text{Uniform}(2, 9), \quad N = 10^{\eta} \tag{S7}$$

$$\theta = 2N\mu\ell \tag{S8}$$

$$\mathbb{E}[T] = \sum_{j=2}^{n} \frac{2}{n_j(n_j - 1)} \tag{S9}$$

$$\mathbb{E}[L] = \sum_{j=2}^{n} \frac{2}{n_j - 1} \tag{S10}$$

$$s \sim \text{Poisson}(\lambda), \quad \lambda = \frac{1}{2}\mathbb{E}[L]\theta \tag{S11}$$

where s represents the sampled value of the number of segregating sites for a value of λ based on the proposed sets of parameters. The value of μ was chosen as the order-of-magnitude estimate for the somatic mutation rate as determined from past studies of the single-cell somatic mutation rate. N is log-uniform distributed from 10^2 to 10^9. The lower bound of the range of population sizes we sampled is the next-lowest order of magnitude of our observed sample size in each brain (560 cells rounded down to 100 cells), while the upper bound is one order of magnitude short of the approximately 85 billion cells within the human brain based on existing estimates (Herculano-Houzel, 2009). The acceptance probability for the parameters estimated above is

$$\text{Accept. Prob.} = \frac{\text{Poisson}(s; \lambda)}{\text{Poisson}(s; S)} \tag{S12}$$

where $\text{Poisson}(\cdot; \lambda)$ refers to the probability mass function of the Poisson distribution with parameter λ. The null model assumes that the number of segregating sites in the population is the same as the observed number of segregating sites S in the sample, which is obtained directly from the genotype matrix.

We conducted 5000 iterations of rejection sampling and retained parameters values with acceptance probability greater than 0.95. The accepted values of Ω can be used to compute posterior estimates to describe the properties of the population that gave rise to our sample and its lineage.

Modifications for estimating the per-variant time-of-origin (TOO)

A modified procedure was conducted to estimate the coalescence times of subpopulations of cells that all share the same variant and, by extension, the time at which the variant arose in the population. We assume that our phylogenetic tree ψ contains a subtree ψ_i for each mutation i that was mapped to a branch and corresponds to the portion of the tree that descends from an ancestral branch to which mutation i is mapped.

We define S_i as the number of segregating sites in ψ_i. Given the parameters that we estimated above, we now seek to estimate additional parameters $\Omega_i = \{A_i, A_m, L_i, T_i, T_{i,m}\}$, where A_m is the number of progenitors of the entire population, A_i is the number of progenitors of the subpopulation, T_i is the time for the subpopulation to coalesce to its MRCA, L_i is the sum of the lengths of all branches of ψ_i, and $T_{i,m}$ is the time between the MRCA of the entire population and the MRCA of the subpopulation, i.e. when the variant originated in the population relative to the time when the whole population's MRCA started to produce the lineage. The values of Ω_i are iteratively inferred until $A_i \leq 1$, i.e., when the subpopulation coalesces to its single MRCA (the lone cell bearing mutation i). Using initial values of $A_i = n_i$, $A_m = n$, and $T_i = 0$,

$$W = \frac{2}{A_m(A_m - 1)} \tag{S13}$$

$$T_i \leftarrow T_i + W \tag{S14}$$

$$L_i \leftarrow L_i + A_i W \tag{S15}$$

$$p = \frac{A_i(A_i - 1)}{A_m(A_m - 1)} \tag{S16}$$

$$A_m \leftarrow A_m - 1 \tag{S17}$$

$$U \sim \text{Bernoulli}(p) \tag{S18}$$

$$A_i \leftarrow A_i - U \tag{S19}$$

$$T_{i,m} = \begin{cases} 0, & \text{if } A_m = 1 \\ \sum_{j=1}^{A_m} W_j, & \text{otherwise, where } W_j \sim \text{Exponential}(\frac{j(j-1)}{2}) \end{cases} \tag{S20}$$

$$T_m = T_i + T_{i,m} \tag{S21}$$

$$s \sim \text{Poisson}(\lambda_i), \quad \lambda_i = \frac{L_i \theta}{2} \tag{S22}$$

Until $A_i \leq 1$, where T_m is the coalescent time of the entire tree and T_i is the coalescent time of the subtree with ancestral mutation i. Here, s is the sampled number of segregating sites in a subpopulation with parameters defined by Ω_i. θ is derived from the estimate of the whole-tree coalescent above, as we assume that the population mutation rate does not change significantly throughout the lineage. Then, the acceptance probability of the parameters Ω_i is

$$\text{Accept. Prob.} = \frac{\text{Poisson}(s; \lambda_i)}{\text{Poisson}(s; S_i)} \tag{S23}$$

Rejection sampling is run for 1000 iterations for each variant.

Conversion of coalescent time parameters to real-world time estimates

The estimated coalescent time parameters $\{T, T_i, T_{i,m}, T_m\}$ must be converted to real-world time units. To do so, we rely upon our previously obtained estimate for N, and we assumed a division rate of 250,000 new cells per minute in the developing brain (Ackerman, 1992). Thus, the number of weeks within any estimate for coalescent time from our procedure is

$$\text{Number of weeks} = \frac{T \times N}{(\log_2 \frac{2.5\times10^5 \text{ new cells}}{60 \text{ seconds}} \times 8.64 \times 10^4 \frac{\text{seconds}}{\text{day}} \times 7 \frac{\text{days}}{\text{week}})} \tag{S24}$$

The $\log_2$ transformation allows for converting the number of new cells per second to the number of new cell divisions per second, as each coalescent event implicitly represents a new cell division event.

Status of single-cell mutations

The coalescent time of a single-cell mutation in our tree is assigned to the total coalescent time of the entire tree as a "censored" estimate of the time-of-origin. The data do not allow us to rule out that the variant is present in additional cells in the overall population from which we took our sample of cells. However, the coalescent time of the whole lineage built on the sample of cells can provide an estimate for the latest time at which single-cell mutations occurred in our sampled cells.

Visualization of variant timelines
The "grid" and "igraph" packages were used to construct the variant timelines, and custom code to produce the timeline plots in **Fig. 4** is provided in the linked code repository.

References

Ackerman, S., 1992. *The Development and Shaping of the Brain*. National Academies Press (US).

Beaumont, M. A., Zhang, W., and Balding, D. J., 2002. Approximate bayesian computation in population genetics. *Genetics*, **162**(4):2025–2035.

Csilléry, K., Blum, M. G. B., Gaggiotti, O. E., and François, O., 2010. Approximate bayesian computation (ABC) in practice. *Trends Ecol. Evol.*, **25**(7):410–418.

Gillespie, J., 1998. *Population Genetics: A Concise Guide*. A Johns Hopkins paperback : Science. Johns Hopkins University Press.

Herculano-Houzel, S., 2009. The human brain in numbers: a linearly scaled-up primate brain. *Front. Hum. Neurosci.*, **3**:31.

Hodge, R. D., Bakken, T. E., Miller, J. A., Smith, K. A., Barkan, E. R., Graybuck, L. T., Close, J. L., Long, B., Johansen, N., Penn, O., *et al.*, 2019. Conserved cell types with divergent features in human versus mouse cortex. *Nature*, **573**(7772):61–68.

Huang, A. Y., Li, P., Rodin, R. E., Kim, S. N., Dou, Y., Kenny, C. J., Akula, S. K., Hodge, R. D., Bakken, T. E., Miller, J. A., *et al.*, 2020. Parallel RNA and DNA analysis after deep sequencing (PRDD-seq) reveals cell type-specific lineage patterns in human brain. *Proceedings of the National Academy of Sciences*, **117**(25):13886–13895.

Kingman, J. F. C., 1982. On the genealogy of large populations. *J. Appl. Probab.*, **19**(A):27–43.

Tavaré, S., Balding, D. J., Griffiths, R. C., and Donnelly, P., 1997. Inferring coalescence times from DNA sequence data. *Genetics*, **145**(2):505–518.

Tran, T. D., Hofrichter, J., and Jost, J., 2013. An introduction to the mathematical structure of the Wright-Fisher model of population genetics. *Theory Biosci.*, **132**(2):73–82.

Wu, Y., 2020. Accurate and efficient cell lineage tree inference from noisy single cell data: the maximum likelihood perfect phylogeny approach. *Bioinformatics*, **36**(3):742–750.
